# Unchanged nitrate and nitrite isotope fractionation during heterotrophic and Fe(II)-mixotrophic denitrification suggest a non-enzymatic link between denitrification and Fe(II) oxidation

**DOI:** 10.3389/fmicb.2022.927475

**Published:** 2022-09-02

**Authors:** Anna-Neva Visser, Scott D. Wankel, Claudia Frey, Andreas Kappler, Moritz F. Lehmann

**Affiliations:** ^1^Aquatic and Isotope Biogeochemistry, Department of Environmental Sciences, Basel University, Basel, Switzerland; ^2^Stable Isotope Biogeochemistry, Marine Chemistry and Geochemistry, Woods Hole Oceanographic Institution, Falmouth, MA, United States; ^3^Geomicrobiology, Center for Applied Geosciences, Eberhard Karls University, Tuebingen, Germany; ^4^Cluster of Excellence: EXC 2124: Controlling Microbes to Fight Infection, Tuebingen, Germany

**Keywords:** denitrification, nitrate/nitrite isotopes, iron oxidation, isotope fractionation, carbon substrate

## Abstract

Natural-abundance measurements of nitrate and nitrite (NO_x_) isotope ratios (δ^15^N and δ^18^O) can be a valuable tool to study the biogeochemical fate of NO_x_ species in the environment. A prerequisite for using NO_x_ isotopes in this regard is an understanding of the mechanistic details of isotope fractionation (^15^ε, ^18^ε) associated with the biotic and abiotic NO_x_ transformation processes involved (e.g., denitrification). However, possible impacts on isotope fractionation resulting from changing growth conditions during denitrification, different carbon substrates, or simply the presence of compounds that may be involved in NO_x_ reduction as co-substrates [e.g., Fe(II)] remain uncertain. Here we investigated whether the type of organic substrate, i.e., short-chained organic acids, and the presence/absence of Fe(II) (mixotrophic vs. heterotrophic growth conditions) affect N and O isotope fractionation dynamics during nitrate (NO_3_^–^) and nitrite (NO_2_^–^) reduction in laboratory experiments with three strains of putative nitrate-dependent Fe(II)-oxidizing bacteria and one canonical denitrifier. Our results revealed that ^15^ε and ^18^ε values obtained for heterotrophic (^15^ε-NO_3_^–^: 17.6 ± 2.8‰, ^18^ε-NO_3_^–^:18.1 ± 2.5‰; ^15^ε-NO_2_^–^: 14.4 ± 3.2‰) vs. mixotrophic (^15^ε-NO_3_^–^: 20.2 ± 1.4‰, ^18^ε-NO_3_^–^: 19.5 ± 1.5‰; ^15^ε-NO_2_^–^: 16.1 ± 1.4‰) growth conditions are very similar and fall within the range previously reported for classical heterotrophic denitrification. Moreover, availability of different short-chain organic acids (succinate vs. acetate), while slightly affecting the NO_x_ reduction dynamics, did not produce distinct differences in N and O isotope effects. N isotope fractionation in abiotic controls, although exhibiting fluctuating results, even expressed transient inverse isotope dynamics (^15^ε-NO_2_^–^: –12.4 ± 1.3 ‰). These findings imply that neither the mechanisms ordaining cellular uptake of short-chain organic acids nor the presence of Fe(II) seem to systematically impact the overall N and O isotope effect during NO_x_ reduction. The similar isotope effects detected during mixotrophic and heterotrophic NO_x_ reduction, as well as the results obtained from the abiotic controls, may not only imply that the enzymatic control of NO_x_ reduction in putative NDFeOx bacteria is decoupled from Fe(II) oxidation, but also that Fe(II) oxidation is indirectly driven by biologically (i.e., *via* organic compounds) or abiotically (catalysis *via* reactive surfaces) mediated processes co-occurring during heterotrophic denitrification.

## Introduction

Nitrate (NO_3_^–^) is a widespread inorganic pollutant with detrimental impacts on ground- and thus drinking water qualities worldwide (World Health Organization, 2011). Nitrate contamination of freshwater resources, which is mostly a result of intense anthropogenic practices (i.e., agricultural application of fertilizers), is partially mitigated by *in situ* biogeochemical processes (Fazal et al., 2003; Rivett et al., 2008; Husic et al., 2019). Particularly the biological transformation of NO_3_^–^ to N_2_, known as denitrification, might play a crucial role in ecosystem resilience. Denitrification is a step-wise enzymatically driven reaction cascade reducing NO_3_^–^ to N_2_ *via* the formation of intermediate nitrite (NO_2_^–^), nitric oxide (NO) and nitrous oxide (N_2_O) under anoxic/suboxic conditions (Knowles, 1982; Cojean et al., 2019). Commonly, this enzymatic reduction of NO_3_^–^ is coupled to the oxidation of an organic substrate (i.e., chemoorganotrophic denitrification; Eq. 1) (Bengtsson and Bergwall, 1995; Zumft, 1997; Calderer et al., 2010). However, as aquifers are mostly oligotrophic (i.e., limited in bioavailable carbon; see Goldscheider et al., 2006), several studies have shown that microbes are frequently able to alternatively couple NO_3_^–^ reduction to the oxidation of inorganic electron donors such as H_2_S or Fe(II) (chemolithotrophic denitrification; Eq. 2) (Zumft, 1997; Torrento et al., 2010; Frey et al., 2014; Wenk et al., 2014). Both, N and Fe cycles involve redox-reactive species and thus, under a variety of environmental conditions (Anderson and Levine, 1986; Kappelmeyer et al., 2003; Kulkarni et al., 2016; Ostrom et al., 2016), existing evidence indicates that NO_2_^–^ can also be reduced chemically by Fe(II) to N_2_O and N_2_ (Jones et al., 2015; Grabb et al., 2017; Visser et al., 2020). This “chemodenitrification” (Eq. 3) has been observed in soils, rice paddy fields and under laboratory conditions, and cross-links both biogeochemical cycles without requiring direct microbial activity.


(1) $$0.84\,C\,H_{3}\,C\,O\,O\,H+N\,O_{3}^{-}\to 0.08\,C_{5}\,H_{7}\,O_{2}\,N+H\,C\,O_{3}^{-}+0.3\,C\,O_{2}+0.92\,H_{2}\,O+0.46\,N_{2}$$

(2) 10Fe+2+2NO3-+24H2O→10Fe(OH)3+N2+18H+

(3) 4Fe2++2NO2-+5H2O→4FeOOH+N2O+6H+

Despite clear evidence for interactions between N and Fe during denitrification, the nature of such interactions and their importance in the natural environment remain poorly understood (Hansel et al., 2015; Berg et al., 2016; Kappler and Bryce, 2017). Fe(II) can interfere with the N cycle in multiple ways, and distinguishing between true chemolithoautotrophic denitrification (biotic; Eq. 2) and chemodenitrification (abiotic; Eq. 3) is challenging. Several studies have demonstrated linkages between the N and Fe cycles through the activity of (putative) nitrate-dependent Fe(II)-oxidizing (NDFeOx) bacteria. However, debate on whether the reaction is indeed enzymatically mediated, i.e., directly linking nitrate reductase activity to a Fe(II) oxidation specific enzyme (e.g., Fe(II) oxidase), remains active. Several studies suggest a truly autotrophic metabolic pathway (enzymatic Fe(II) oxidation *via* specific enzymes/Fe(II) oxidation related protein complexes) for NDFeOx bacteria under anoxic conditions (Straub et al., 2004; Su et al., 2015; Laufer et al., 2016; Tominski et al., 2018a; Tian et al., 2020; Jakus et al., 2021; Huang et al., 2022). Conversely, others proposed that the observed Fe(II) oxidation by NDFeOx bacteria is the result of an abiotic side reaction between Fe(II) and the heterotrophically produced intermediate NO_2_^–^ (Carlson et al., 2012; Picardal, 2012; Klueglein et al., 2014). This is also supported by the fact that for most genera of putative NDFeOx bacteria grown under carbon co-substrate and Fe(II)-replete (i.e., mixotrophic) conditions, genomic analyses has not revealed evidence for an enzymatically mediated reaction (Byrne-Bailey et al., 2010; Barco et al., 2015; Ishii et al., 2016; He et al., 2017; Price et al., 2018). Despite the lack of a specific Fe(II) oxidation-related enzyme (i.e., Fe(II) oxidase), *c*-type cytochromes, which are present in most putative NDFeOx bacteria (e.g., He et al., 2017), have been suggested to (in-) directly couple Fe(II) oxidation to nitrate reduction in these strains (Weber et al., 2006; Ilbert and Bonnefoy, 2013). Yet, the mechanistic details of this possible link appear to be rather complex and have, to date, not been investigated (e.g., Liu et al., 2018, 2019).

Natural-abundance measurements of NO_3_^–^ and NO_2_^–^ (NO_x_) isotope ratios (δ^15^N and δ^18^O) can potentially be used to disentangle different N turnover processes (Granger et al., 2008; Sigman et al., 2009; Jones et al., 2015). The dual NO_3_^–^/ NO_2_^–^ isotope approach is based on the premise that specific N-transformation reaction mechanisms (abiotic or biotic) are associated with more or less characteristic N- vs. O isotope partitioning. In general, during the reduction of both NO_3_^–^ and NO_2_^–^, the lighter N and O isotopologues are preferably consumed and the substrate pool becomes enriched in the heavier isotopes (i.e., ^15^N, ^18^O). The kinetic N and O isotope effects during heterotrophic NO_3_^–^/NO_2_^–^ reduction primarily arise as a function of the enzyme involved, but also strongly depend on reaction kinetics (Granger et al., 2008; Asamoto et al., 2021). Reaction kinetics, in turn, are controlled by environmental factors, such as temperature, cell density, growth rate, substrate type and/or concentration (Bryan et al., 1983; Needoba et al., 2004; Kritee et al., 2012; Wunderlich et al., 2012; Karsh et al., 2014; Martin and Casciotti, 2016). Enzymes mediating NO_3_^–^ and NO_2_^–^ reduction are usually located within the peri- or the cytoplasm of the cell, and differential limitation of the cellular uptake (and efflux) of NO_3_^–^ or NO_2_^–^, affects the expression of N and O isotope fractionation at the ecosystem level (Granger et al., 2008). To this end, organic substrate availability may affect the reaction kinetics of heterotrophic NO_x_ reduction, leading to a change in stable isotope fractionation (Bryan et al., 1983; Kritee et al., 2012; Martin and Casciotti, 2016). In addition, NO_3_^–^/NO_2_^–^ reduction rates differ depending on the associated electron donor (e.g., carbon source) (Devlin et al., 2000; Di Capua et al., 2019; Li et al., 2022), which not only depend on the redox differential between the redox couple, but possibly also on the different uptake mechanisms involved. For example, acetate “uptake” is considered to be controlled by passive diffusion, while for the uptake of succinate, the TRAP transporter [C(4)-dicarboxylate ABC transporter] is responsible for active transport into the cell of many bacteria (Jolkver et al., 2009; Groeneveld et al., 2010; Valentini et al., 2011). Thus, the compound-specific properties of the organic matter involved can ultimately affect the internal cellular substrate pool available for enzymatic NO_3_^–^/NO_2_^–^ reduction, possibly modulating the partitioning of energy resources, which may change expression of an organism-level N and/or O isotope fractionation. Moreover, limitation and changes in the compound-specific properties of the organic substrate may induce a switch from chemoorganotrophic to chemolithotrophic denitrification (Muehe et al., 2009; Bryce et al., 2018; Peng et al., 2018), and/or may lead to metabolic bottlenecks and the accumulation of intermediates such as NO_2_^–^ (Weber et al., 2006; Carlson et al., 2013; Klueglein and Kappler, 2013). This, in turn, will affect denitrification rates (Devlin et al., 2000; Hosono et al., 2015; Di Capua et al., 2019; Li et al., 2022) and thus, should ultimately be reflected in N and O isotope fractionation patterns. Finally, if grown under mixotrophic conditions at relatively high Fe(II) concentrations, putative NDFeOx bacteria have the tendency to become encrusted with Fe(III) (oxyhydr)oxides, and cellular substrate uptake may partially be hindered (Kappler et al., 2005; Schädler et al., 2009; Klueglein et al., 2014; Chen et al., 2020). Both, abiotic reduction of biologically produced NO_2_^–^ with Fe, and/or Fe(III) (oxyhydr)oxide encrustation at the cellular level are thus expected to produce NO_3_^–^ and NO_2_^–^ dual isotope signatures that are different from those of canonical or chemolithotrophic denitrification (Buchwald et al., 2016; Grabb et al., 2017). In this context, coupled N and O isotope measurements promise to shed light on the mode of denitrification, possible links to Fe(II) oxidation, and potentially on the environmental relevance of chemodenitrification (e.g., in Fe-rich, reducing environments).

Thus far, most NO_x_ isotope fractionation studies have focused on chemoorganotrophic denitrification with either NO_3_^–^ or NO_2_^–^ as the initial substrate (Bryan et al., 1983; Granger et al., 2008; Sovik and Morkved, 2008; Kritee et al., 2012; Martin and Casciotti, 2016). Studies on the isotope effects of chemolithotrophic denitrification are rare (Chen et al., 2020; Margalef-Marti et al., 2020) or limited to sulfidic electron donors (Frey et al., 2014; Wenk et al., 2014). Similarly, to our knowledge, only a few studies exist that have investigated the dual NO_2_^–^ N and O isotope effects associated with Fe(II) coupled chemodenitrification (Jones et al., 2015; Buchwald et al., 2016; Grabb et al., 2017; Visser et al., 2020; Chen et al., 2021). To date, no direct comparison of the NO_3_^–^/NO_2_^–^ isotope effects focussing on the apparent association of denitrification with Fe(II) oxidation, as well as a possible influences caused by varying the organic carbon source, has been performed. Yet, directly or indirectly, the involvement of Fe(II) may represent an important control on the dual isotopic composition of NO_3_^–^/NO_2_^–^ in denitrifying environments.

Here, we tested the N and O isotope dynamics during NO_3_^–^/NO_2_^–^ reduction by denitrifying bacterial strains that have previously been linked to the oxidation of Fe(II). Our specific goals were to calibrate the dual NO_3_^–^ and NO_2_^–^ isotope systematics associated with denitrification by putative NDFeOx bacteria under different carbon-substrate conditions, as well as to understand the modulating role that the presence of Fe(II) may have on net N and O isotope effects. In turn, the combined geochemical and isotope evidence gained from our experiments aids our efforts to use NO_3_^–^/NO_2_^–^ dual isotope ratios for constraining the interaction (biotic vs. abiotic) between denitrification and Fe(II) oxidation, and thus to verify the metabolic lifestyle (heterotrophic or mixotrophic) of the studied microorganisms.

## Materials and methods

### Microorganisms

The facultative anaerobe *Acidovorax* sp. strain BoFeN1, a chemoorganotrophic, nitrate-reducing, Fe(II)-oxidizing microorganism, was originally isolated from Lake Constance sediments (Kappler et al., 2005). *Acidovorax delafieldii* strain 2AN is closely related to strain BoFeN1 and was isolated from an iron-rich river sediment in Wisconsin, United States (Chakraborty et al., 2011). “*Pseudogulbenkiania ferrooxidans”* strain 2002 was isolated from a freshwater lake in Illinois, United States, and cultures were obtained from the Deutsche Sammlung von Mikroorganismen und Zellkulturen (DSMZ), Braunschweig, Germany (Byrne-Bailey and Coates, 2012). *Paracoccus denitrificans* (here strain ATCC 19367), originally isolated by Beijernick in 1990 from soil (ATCC, 2016), is used to represent the canonical denitrifiers, and serves here as a control. Based on genome analysis, all strains harbor the genes to express the respiratory nitrate reductase (Nar) and the heme (cd_1_NIR)-containing nitrite reductase (NirS) (Supplementary Table 1). Genomic evidence for strain 2002 also indicates the presence of a *napA* encoding gene (Byrne-Bailey et al., 2012). In addition, the draft genome sequence of BoFeN1 indicates the presence of an additional Cu-containing nitrite reductase (*nirK*) gene (Gauger, 2016; Price et al., 2018). To date, no genetic evidence for enzymatically mediated Fe(II) oxidation, i.e., the presence of a Fe(II) oxidase or a similar protein complex that directly couples nitrate reduction to Fe(II) oxidation, by these microorganisms has been identified (Byrne-Bailey et al., 2012; Ishii et al., 2016; Price et al., 2018).

### Medium preparation and cultivation conditions

#### Anoxic solutions

All solutions were prepared under sterile anoxic conditions. MilliQ water was heated until boiling and cooled under continuous flushing with N_2_ gas. A 1M Fe(II)Cl_2_ solution was prepared by dissolution in anoxic MilliQ water while flushing with N_2_ gas. Afterward, the solution was filter-sterilized (22 μm) into a sterile, N_2_-flushed serum bottle. The same procedure was used for preparation of a 1 M NaNO_2_ stock solution. Solutions of NaNO_3_, NaCH_3_COOH (Na-acetate) and C_4_H_6_O_4_ (succinate) were prepared similarly using autoclave sterilization.

#### Cultivation conditions

Previous cultivation studies involving NDFeO have distinguished between autotrophic (assimilating CO_2_), heterotrophic (using C from an organic substrate for biosynthesis) and mixotrophic (utilizing both metabolic pathways) growth conditions (Nordhoff et al., 2017; Tominski et al., 2018a). For example, strains BoFeN1, 2AN and 2002 have been referred to as mixotrophic NDFeOx bacteria (Kappler et al., 2005; Chakraborty et al., 2011; Klueglein et al., 2014). Yet, in previous studies, these strains have always been cultivated in the presence of CO_2_ (headspace) and an organic acid (medium), providing the possibility for mixotrophy. Therefore, the term “mixotrophic” could be misleading, as it has been used to refer to growth conditions rather than to the actual active metabolic pathways. Thus far, actual autotrophic growth has only been demonstrated for three *Gallionellaceae*-bearing enrichments cultures (Tominski et al., 2018a; Huang et al., 2022), as well as a enrichment culture KS-like consortia obtained from e.g., activated sludge (Tian et al., 2020). Here we use the term “mixotrophic” (vs. heterotrophic) growth in an operational context, to distinguish between cultivation in the presence or absence of Fe(II), not as an indication of a specific metabolic activity.

#### Medium preparation

An anoxic 22 mM bicarbonate-buffered low-phosphate medium [1.03 mM KH_2_PO_4_, 3.42 mM NaCl, 5.61 mM NH_4_Cl, 2.03 mM MgSO_4_⋅7 H_2_O and 0.68 mM CaCl_2_⋅2 H_2_O; 1 ml 7 vitamin solution (Widdel and Pfennig, 1981)/1 ml SL-10 trace element solution per liter medium (Widdel et al., 1983)] with a N_2_/CO_2_ (90/10 v/v) headspace was used for the cultivation of all strains. The media were prepared in a Widdel flask under sterile conditions, and while flushing with N_2_/CO_2_ gas. Substrates were added after pH adjustment (pH 7.1). For nitrate-based experiments, ∼0.8 mM NaNO_3_ and ∼0.5 mM acetate/succinate were added to the Widdel flask directly. Nitrite-based experiments were conducted with ∼0.2 mM NaNO_2_ and ∼0.1 mM acetate/succinate. 25 ml of medium were dispensed anoxically in 50 ml heat-sterilized (oven, 4 h, 180°C) serum bottles. For Fe-amended experiments, the Fe-free medium was first dispensed without any electron acceptors/donors in Schott bottles, then ∼0.8 mM (∼0.2 mM for nitrite-based experiments) Fe(II)Cl_2_ solution was added. To enhance precipitate formation, the Schott bottles were stored in the dark at 4°C. Prior to further aliquoting the Fe-containing medium inside an anoxic glove box (MBraun, N_2_ 100%), the other substrates were added as described above. The medium was stirred continuously while dispensing it equally into 50 ml serum bottles (25 ml each). The bottles were closed with autoclaved butyl rubber stoppers, and crimp-sealed.

### Incubation experiments and sampling

In total 16 nitrate-based and 10 nitrite-based experiments were conducted. Half of the experiments were amended with acetate or succinate, respectively. To eight of the nitrate-based experiments, Fe(II) was added. For each sampling time point (between 0 and up to 50 h), nine replicates were sacrificed. All serum bottles were inoculated under sterile conditions with a 4% (v/v) bacterial inoculum (from 25 ml pre-culture grown in the absence of Fe(II), ca. 2.5 × 10^5^ cells/ml), and incubated at 28°C in the dark throughout the experiments. For the abiotic NO_2_^–^ experiment (w/o cells), the same medium was used and the same sacrificial sampling method (nine replicates per time point) was applied. Furthermore, the abiotic experiment was conducted for 30 days and at ∼2 mM NO_2_^–^ and ∼2 mM Fe(II) to enhance reaction dynamics. Hence, the abiotic experiment is not used for direct comparison but rather to discern reaction-dynamic-relevant patterns.

At each sampling time point, replicate serum bottles were transferred to the anoxic glove box. There, using a 20 ml syringe, the headspace was quantitatively transferred into He-purged 12 ml Exetainer vials (LABCO) for subsequent gas analysis. After shaking the serum bottle thoroughly, 5 ml liquid were transferred *via* sterile filtration (0.22 μm) into a 5 ml Eppendorf tube. For isotope analysis, sterile-filtered samples were either directly transferred to 12-ml glass vials (for NO_2_^–^ analysis with the azide method, see section “N and O isotope analysis in nitrate and nitrite”) or treated first with 40 mM amidosulfonic acid (SFA, to remove any traces of NO_2_^–^ prior to nitrate analysis by the denitrifier method, see section “N and O isotope analysis in nitrate and nitrite”) and stored at –20°C until measurement (see Granger and Sigman, 2009). For Fe analysis, a 1 ml sample aliquot was transferred into a 1 ml Eppendorf tube and centrifuged for 5 min at 12,100 rcf (Eppendorf, MiniSpin). 200 μl of supernatant were taken and diluted 1:5 with 40 mM SFA (NO_2_^–^ removal prior to analysis) for the ferrozine assay (Stookey, 1970; Klueglein and Kappler, 2013). The pellet was dissolved in 1 ml of 1 M HCl (for Fe_total_, by ferrozine analysis). SFA- and/or HCl-fixed samples were stored in the dark at 4°C until measured. For organic substrate concentration measurements, 500 μl of the filtered sample were stored at 4°C in the dark until measured.

### Concentration analyses

A continuous-flow analyser (CFA, Seal Analytics AA3) was used for determining NO_3_^–^/NO_2_^–^ concentrations. The method is based on the Griess reaction for the spectrophotometric detection of NO_2_^–^ at 540 nm (López Pasquali et al., 2007; Irandoust et al., 2013). 200 μl of the sterile filtered sample was diluted 1:5 in anoxic MilliQ water and measured within 1 h after sampling. Organic substrate concentrations were analyzed using high-pressure liquid chromatography (HPLC; Shimadzu) with a column to detect organic acids (Column HPX 87H Bio-Rad, RID and DAD 210 nm). The ferrozine assay was performed outside the glovebox using a 96 well plate reader (Thermo Scientific Multiskan GO), where Fe(II) concentrations were determined by absorption spectrophotometry at λ = 562 nm. In Fe-amended experiments, concentrations were determined in all samples, whereas for controls (no Fe), the ferrozine assay was applied only at the beginning and end of the experiment (data not shown). Total Fe(II) concentrations presented are the sum of the $F\,e_{a\,q}^{2+}+F\,e\,{\left(I\,I\right)}_{p\,e\,l\,l\,e\,t}$concentrations. For the determination of N_2_O concentrations in the gas phase, triplicate gas samples were diluted 1:5 with 5.0 He-gas into He-pre-purged 12 ml Exetainer vials and sent to the University of Zürich, where N_2_O was quantified *via* GC-MS analysis (Agilent 7,890 with micro-ECD und FID; Column Porapak Q 80/100) (see Niklaus et al., 2016). The N_2_O concentrations presented in the graphs refer to total N_2_O in the liquid, based on the analysis of the gas phase in the headspace of a given volume in the closed bottle. Total N_2_O concentrations were calculated using the Henry constant (K_H_ = (p_gas_ [atm]/C [mol/l]) = [1*atm/mol]) (Balsiger, 2001; Sander et al., 2022). Assuming that liquid and gas phase were in equilibrium, the total concentration in the liquid phase was calculated according to n_solution_ = n_gas_*[(RT/K_H_)(V_solution_/V_gas_)], whereas n represents the number of moles in solution, R is the universal gas constant, in air (J/mol*K) = 8.314, T the temperature (298.15 K), K_H_ Henry constant, and V the respective volumes (Balsiger, 2001).

### N and O isotope analysis in nitrate and nitrite

For the analysis of N and O isotope ratios in NO_3_^–^, the “denitrifier method” was applied (Sigman et al., 2001). Briefly, NO_3_^–^ is microbially converted to N_2_O by a culture of *P. aureofaciens*, which is then purified and analyzed using a modified purge-and-trap system coupled to a CF-IRMS (Thermo Scientific IRMS Delta V) (McIlvin and Casciotti, 2010). Blank contribution was generally lower than 0.3 nmol (as compared to 20 nmol of sample). Oxygen isotope exchange with H_2_O during the reduction of NO_3_^–^ to N_2_O was corrected for, and was never higher than 4%. Isotope values were calibrated by standard bracketing using internal and international NO_3_^–^ isotope standards with known N and O isotopic composition, namely IAEA-N3 (δ^15^N: +4.7 ± 0.2‰, δ^18^O: +25.6 ± 0.4‰), USGS32 (δ^15^N: +180‰, δ^18^O: 25.7 ± 0.2‰), USGS34 (δ^15^N: –1.8 ± 0.1‰, δ^18^O: –27.9 ± 0.3‰). For analysis of N and O isotope ratios in NO_2_^–^, a slightly adapted version of the “azide method” was applied (McIlvin and Altabet, 2005), as previously described in Visser et al. (2020). NO_2_^–^ isotope standards, namely N-7373 (δ^15^N: –79.6‰, δ^18^O: +4.5‰) and N-10219 (δ^15^N: +2.8‰; δ^18^O; +88.5‰) (Casciotti and McIlvin, 2007) were prepared freshly for each sampling time point and processed similarly. NO_3_^–^ and NO_2_^–^ N and O isotope data are expressed using the common delta notation and reported as per mille deviation (‰) relative to AIR-N_2_ and VSMOW, respectively (δ^15^*N* = ([^15^N]/[^14^N])_sample_ /[^15^N]/[^14^N]_air_N2_ - 1) × 1,000) and δ^18^O = ([^18^O]/[^18^O]_sample_ /[^18^O]/[^16^O]_VSMOW_ - 1) × 1,000). Analytical precision for NO_3_^–^ δ^15^N and δ^18^O, based on replicate measurements of laboratory standards and samples was ± 0.2‰ and ± 0.4‰ (1 SD), respectively. The analytical precision for NO_2_^–^ δ^15^N and δ^18^O was ± 0.4‰ and ± 0.6‰ (1 SD), respectively.

The enrichment factor, or isotope effect ε, is calculated according to the simplified Rayleigh distillation equation (Mariotti et al., 1981) for a closed system:


(4) $$\delta_{t}=\delta_{0}-\varepsilon \,l\,n\,\left(\frac{C_{t}}{C_{0}}\right)$$

Where δ (δ^15^N or δ^18^O) represents the N or O isotopic composition of the substrate (e.g., nitrate or nitrite) at any given time point t and at the beginning of the experiment (t_0_), respectively. C_t_ refers to the substrate concentration at time t, while C_0_ refers to the initial concentration. In a Rayleigh diagram, where δ^15^N or δ^18^O are plotted against the residual fraction of the substrate, the slope of the regression line approximates the N and O isotope effects (^15^ε or ^18^ε), respectively.

## Results

### Nitrate as electron acceptor—heterotrophic vs. mixotrophic growth

#### Substrate consumption and intermediate production

**FIGURE 1:**
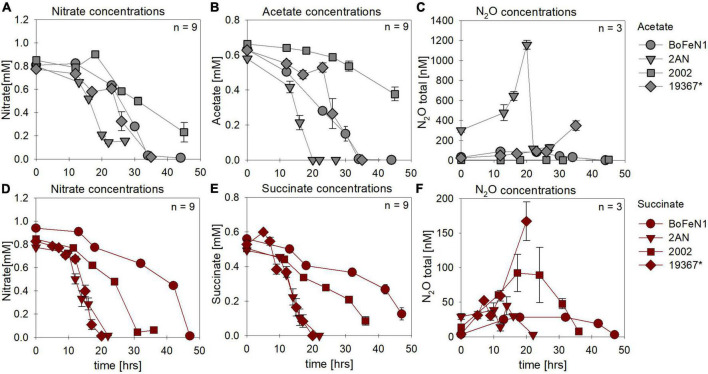
Concentrations of nitrate **(A,D)** and organic acids [acetate (gray), succinate (red)] consumed **(B,E)** and N_2_O produced **(C,F)** during heterotrophic growth in strains of *Acidovorax* sp. strain BoFeN1, *Acidovorax delafieldii* strain 2AN, “*Pseudogulbenkiania ferrooxidans”* strain 2002 and the control denitrifier (*) *Paracoccus denitrificans* ATCC 19367. Note that, for N_2_O concentrations, different axis scales are applied. Standard error calculated from biological replicates (*n* = 9, 3) is represented by the error bars.

Under heterotrophic growth conditions, all four strains were amended with NO_3_^–^ and acetate/succinate. No systematic differences in both NO_3_^–^ reduction (Figures 1A,D and Table 1) and organic acid oxidation (Figures 1B,E) were discernible, and NO_3_^–^ reduction (and organic acid oxidation) was, in most cases, nearly complete. Overall, average total consumption between acetate- (acetate: –0.6 ± 0.06 mM; NO_3_^–^: –0.7 ± 0.03 mM) and succinate- (succinate: –0.5 ± 0.02 mM; NO_3_^–^: –0.8 ± 0.04 mM) amended setups, yielded similar results (also see Table 1). If at all, the amendment of succinate resulted in a slightly shorter lag phase (∼5 h). Moreover, in the presence of succinate, denitrification-associated N_2_O production appeared to be slightly enhanced in all strains (Figures 1C,F and Table 1), with the exception of strain 2AN grown on acetate. NO_2_^–^ concentrations remained low throughout the experiments and NO_2_^–^ accumulation was not detected (data not shown). Overall, no distinct difference between either acetate- or succinate-amended setups was observed, indicating that reaction dynamics of NO_3_^–^ reduction, organic acid oxidation, as well as N_2_O production, were indistinguishable.

**TABLE 1:** Nitrate-based experiments in the presence and absence of Fe(II); – for reduction, + for production.

Strain	Org. substrate	[mM]	NO_3_^–^[mM]	NO_2_^–^[mM]*	N_2_O [μM]*	Fe(II) [mM]
*Acidovorax* sp. strain BoFeN1	Acetate	–0.66 ± 0.02	–0.64 ± 0.06	+0.01 ± 5 × 10^–4^	+0.24 ± 0.001	–0.27 ± 0.03
	Succinate	–0.57 ± 0.002	–0.60 ± 0.08	+0.03 ± 1.1 × 10^–4^	+0.25 ± 0.07	–0.75 ± 0.004
	Acetate	–0.63 ± 0.006	–0.8 ± 0.06	+0.01 ± 4 × 10^–4^	+0.06 ± 3.9x10^–4^	H
	Succinate	–0.44 ± 0.04	–0.93 ± 0.13	+0.003 ± 7.9 × 10^–5^	+0.02 ± 4.5 × 10^–4^	H
*Acidovorax delafieldii* strain 2AN	Acetate	–0.61 ± 0.08	–0.7 ± 0.04	+0.001 ± 2.7 × 10^–4^	+72.4 ± 1.6	–0.34 ± 0.04
	Succinate	–0.5 ± 0.008	–0.76 ± 0.09	+0.001 ± 4 × 10^–4^	+0.03 ± 0.005	–0.44 ± 0.03
	Acetate	–0.58 ± 0.007	–0.64 ± 0.07	+0.008 ± 0.001	+0.85 ± 0.08	H
	Succinate	–0.5 ± 0.02	–0.76 ± 0.009	+0.005 ± 1.8 × 10^–4^	+0.54 ± 0.3	H
*“Pseudogulbenkiania ferrooxidans”* strain 2002^†^	Succinate	–0.46 ± 0.02	–0.68 ± 0.08	+0.002 ± 1.3 × 10^–4^	+1.41 ± 0.2	–0.06 ± 0.009
	Acetate	–0.29 ± 0.04	–0.62 ± 0.11	+0.53 ± 0.1	+0.003 ± 6 × 10^–4^	H
	Succinate	–0.42 ± 0.03	–0.78 ± 0.11	+0.001 ± 2.2 × 10^–4^	+0.08 ± 0.03	H
*Paracoccus denitrificans* ATCC 19367^†^	Acetate	–0.63 ± 0.007	–0.76 ± 0.05	+0.18 ± 0.02	+3.53 ± 0.12	H
	Succinate	–0.53 ± 0.14	–0.82 ± 0.24	+0.28 ± 0.11	+0.16 ± 0.1	H

^†^No growth observed under mixotrophic growth conditions; H heterotrophic growth = no iron amended.

Values are given as mean concentration consumed or produced ± standard error. Consumption values are calculated by ${\bar{X}}_{t\,0}-{\bar{X}}_{t\,e\,n\,d}$, whereas the production values of intermediate species reflect the max. concentration observed $({\bar{X}}_{t,m\,a\,x}-{\bar{X}}_{t\,0}$), and are marked with *.

**FIGURE 2:**
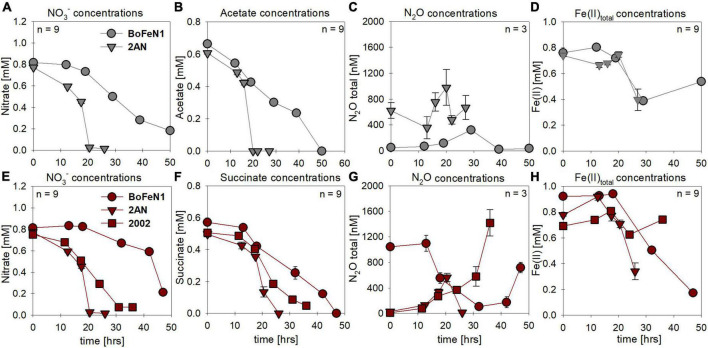
Concentrations for nitrate **(A,E)** and organic acids [acetate (gray), succinate (red)] consumed **(B,F)**, N_2_O produced **(C,G)** and Fe(II) oxidized **(D,H)** during mixotrophic growth in strains of *Acidovorax* sp. strain BoFeN1, *Acidovorax delafieldii* strain 2AN and “*Pseudogulbenkiania ferrooxidans”* strain 2002. No growth was observed for the control denitrifier *Paracoccus denitrificans* strain ATCC 19367, as well as for strain 2002 on acetate. Standard error calculated from biological replicates (*n* = 9, 3) is represented by the error bars.

Under mixotrophic growth conditions (i.e., Fe(II) + organic acid), not all strains showed the ability to reduce NO_3_^–^ (Figures 2A,E). For example, no growth was observed for “*Pseudogulbenkiania ferrooxidans”* strain 2002 cultivated on Fe(II) and acetate (data not shown), and only for the two *Acidovorax* strains, NO_3_^–^ reduction and partial Fe(II) oxidation was observed (Figures 2A,D). Furthermore, N_2_O production was slightly increased in the presence of Fe(II) (Figures 2C,G), and, seemed to have accumulated to a greater degree in succinate-amended setups (Table 1). Again, strain 2AN grown on acetate exhibited a slightly enhanced N_2_O accumulation (Figure 2C vs. Figure 2G, compare to Figure 1C). In contrast, in parallel setups with succinate, all three putative NDFeOx strains performed NO_3_^–^ reduction and Fe(II) oxidation (Figures 2E–H). Fe(II) oxidation and the consumption of the organic substrate occurred simultaneously. Furthermore, lower NO_2_^–^ levels, as well as the slightly elevated N_2_O concentrations, compared to heterotrophic conditions, were observed (Table 1). Yet, the direct comparison between the organic acid treatments (i.e., acetate vs. succinate), based on the maximum consumption (see Table 1) calculated for each setup (heterotrophic vs. mixotrophic), revealed similar trends.

#### N and O isotope dynamics during nitrate reduction

**FIGURE 3:**
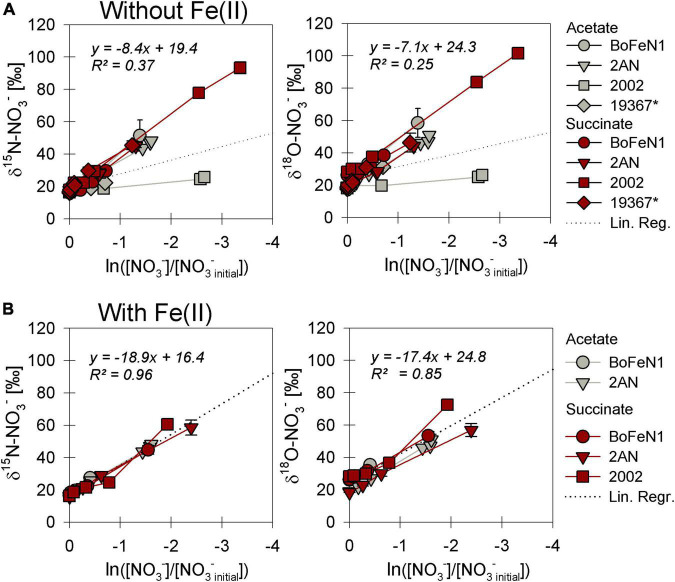
Rayleigh plots of δ^15^N and δ^18^O-NO_3_^–^ for strains grown under heterotrophic **(A)** and mixotrophic **(B)** conditions. The denitrifier control *Paracoccus denitrificans* ATCC 19367 (*) did not grow in the presence of Fe(II) and is not represented in panel **(B)**. Linear regression lines, the slope of which approximate the N and O isotope effects ε for nitrate reduction (Eq. 4), are calculated including all data in plot. Error bars represent standard error calculated from biological replicates (see Table 2).

As NO_3_^–^ consumption proceeded, δ^15^N- and δ^18^O-NO_3_^–^ values increased (Figure 3). When grown under heterotrophic growth conditions, N and O isotope dynamics were more variable (Figure 3A). The N and O isotope effects ranged between 8.4–25.4‰ and 15.9–23.6‰ for N and O, respectively (Table 2). In some heterotrophic experiments (e.g., with strain 2002), acetate gave rise to lower ^15^ε- and ^18^ε-NO_3_^–^ values compared to amendments with succinate. Hence, ^15^ε_total_ (8.4‰, *R*^2^ = 0.4) and ^18^ε_total_ (7.1‰, *R*^2^ = 0.3) values calculated for all setups were rather low. In some cases, data were not easily fit to a closed system Rayleigh model, which may reflect that isotope effects were either not constant, not singular (e.g., more than one process at work) or not irreversible under the conditions of these incubations—all requirements of the Rayleigh model (e.g., BoFeN1 and 2AN on succinate). In these cases, ^15^ε and ^18^ε values are reported based on the linear trends in the Rayleigh plot and thus n values (i.e., time points used for calculation) vary between the setups (see Table 2).

**TABLE 2:** Fractionation factors and *p*-values calculated for the different strains grown on NO_3_^–^ and either acetate or succinate, and in the presence/absence of Fe(II) ± standard error.

Strain	Org. substrate	^15^ε [‰]	*p*-value	*R* ^2^	^18^ε [‰]	*p*-value	*R* ^2^	n
**Presence of Fe(II)**								
*Acidovorax* sp. strain BoFeN1	Acetate	24.3 ± 0.6	0.02	0.99	22.2 ± 0.3	9 × 10^–4^	0.99	4
	Succinate	17.6 ± 0.8	0.004	0.99	16.9 ± 0.7	8 × 10^–4^	0.99	6
*Acidovorax delafieldii* strain 2AN	Acetate	19.2 ± 0.7	0.002	0.99	18.9 ± 1.2	0.002	0.99	6
	Succinate	17.5 ± 0.9	0.04	0.99	15.9 ± 0.9	0.04	0.99	4
*“Pseudogulbenkiania ferrooxidans”* strain 2002^†^	Succinate	22.5 ± 4.9	0.004	0.95	23.4 ± 4.9	0.01	0.95	5
**Absence of Fe(II)**								
*Acidovorax* sp. strain BoFeN1	Acetate	25.4 ± 1.7	0.04	0.99	23.6 ± 1.6	0.02	0.99	4
	Succinate	18.5 ± 1.3	0.001	0.96	17.2 ± 1.2	3 × 10^–4^	0.96	5
*Acidovorax delafieldii* strain 2AN	Acetate	19.2 ± 0.7	0.002	0.99	18.9 ± 1.2	0.002	0.99	6
	Succinate	20.7 ± 1.3	0.005	0.99	18.6 ± 1.4	0.003	0.98	5
*“Pseudogulbenkiania ferrooxidans”* strain 2002	Acetate	*3.04 ± 1.0*	*8.4*× *10^–4^*	*0.96*	*2.7 ± 0.8*	*0.002*	*0.96*	*5*
	Succinate	22.8 ± 1.8	0.02	0.99	22.3 ± 1.8	0.01	0.99	6
*Paracoccus denitrificans* ATCC 19367*	Acetate	*8.4 ± 0.5*	*0.001*	*0.98*	*21.2 ± 2.7*	*0.008*	*0.91*	*4*
	Succinate	22.8 ± 1.9	9.5 × 10^–4^	0.97	22.1 ± 1.64	6 × 10^–4^	0.98	7

F-test obtained p-value testing validity of the ε isotope effect (p < 0.05).

*Canonical denitrifier used as control.

^†^No growth observed under mixotrophic growth conditions + acetate.

ε-values marked italic indicate lowest values based on a shallow increase in δ values but comparable substrate depletion (Rayleigh plot).

**FIGURE 4:**
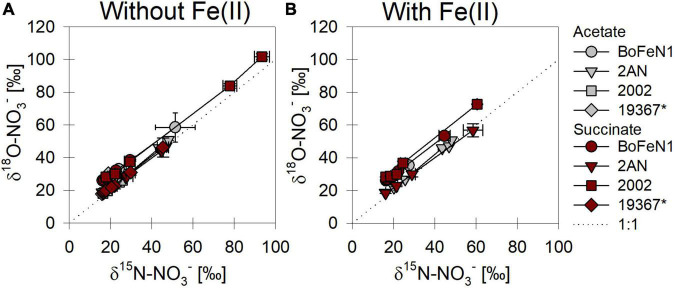
δ^15^N vs. δ^18^O-NO_3_^–^ plots for all strains grown under heterotrophic **(A)** and mixotrophic conditions **(B)**. *Marks the control (denitrifying strain of *Paracoccus denitrificans*). Dashed line indicates a 1:1 relationship.

When grown under mixotrophic growth conditions, i.e., in the presence of Fe(II), N and O isotope fractionation dynamics were more consistent, resulting in very similar trends in δ^15^N- and δ^18^O-NO_3_^–^ values, regardless of the organic acid supplied (Figure 3B). Here, across all treatments and independent of the bacterial strains, NO_3_^–^ isotope fractionation was quite reproducible with average isotope effects of ^15^ε_total_ = 18.9‰ (*R*^2^ = 0.96) and ^18^ε_total_ = 17.4‰ (*R*^2^ = 0.85, see also Table 2), and very similar to the higher-end isotope effect estimates for the heterotrophic setups (^15^ε = 17.6‰ and ^18^ ε = 18.3‰). In addition, observed patterns in coupled δ^15^N- NO_3_^–^ vs. δ^18^O-NO_3_^–^ systematics were very similar for the two experiments (Figure 4). More specifically, neither the absence (Figure 4A)/presence (Figure 4B) of Fe(II), nor the form of organic acid as main or co-substrate, had a distinct impact on coupled N and O isotope fractionation, with all cases exhibiting a coupled N vs. O isotope enrichment following a Δδ^15^N:Δδ^18^O line near 1:1, consistent with previous reports on enzyme-driven denitrification by Nar (Granger et al., 2008; Treibergs and Granger, 2017). Although growth performance of the canonical denitrifier strain ATCC 19367 was inhibited under mixotrophic growth conditions, presence of Fe(II), and under heterotrophic growth conditions, acetate-amendment resulted in lower ^15^ε (∼8.4‰) values, the overall reaction dynamics exhibited similar patterns as the putative NDFeOx strains, indicating that similar processes are at work.

### Nitrite as electron acceptor—heterotrophic vs. mixotrophic growth

#### Substrate consumption and intermediate production

**FIGURE 5:**
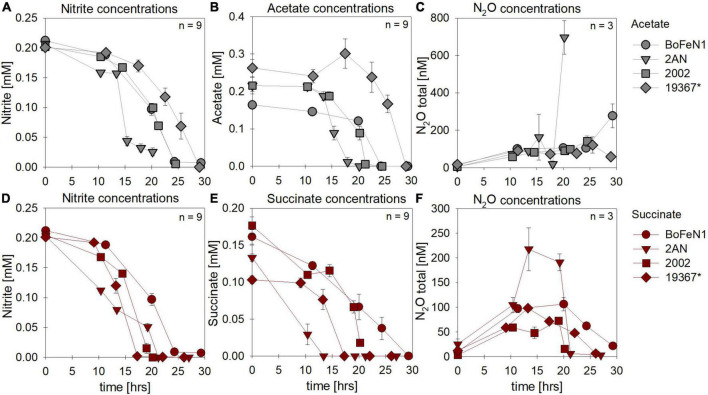
Concentrations for nitrate **(A,E)** and organic acids [acetate (gray), succinate (red)] consumed **(B,F)**, N_2_O produced **(C,G)** and Fe(II) oxidized **(D,H)** during mixotrophic growth in strains of *Acidovorax* sp. strain BoFeN1, *Acidovorax delafieldii* strain 2AN, “*Pseudogulbenkiania ferrooxidans”* strain 2002 and the control denitrifier (*) *Paracoccus denitrificans* ATCC 19367. Note that for organic acid and N_2_O concentrations different axis scales are applied. Error bars represent standard error calculated from biological replicates (*n* = 9, 3).

In most batch cultures grown heterotrophically, NO_2_^–^ and the organic acid were completely consumed by the end of the experiment, regardless of the organic acid provided (Figures 5A,B vs. D,E). Similar to the experiments with NO_3_^–^ as electron acceptor, the lag phase was ∼5 h shorter in the presence of succinate. N_2_O production and consumption was comparable in both setups (Figures 5C,F), yet in the presence of acetate, both *Acidovorax* strains appeared to exhibit N_2_O accumulation rather than the general “production and consumption” pattern observed in the other strains.

**FIGURE 6:**
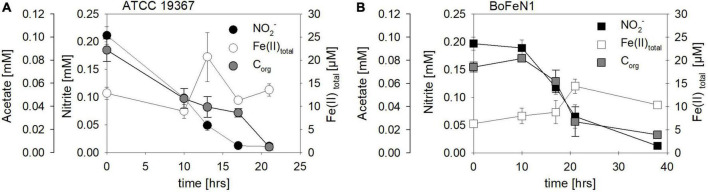
Concentrations of NO_2_^–^ (●, □), Fe(II)_to*tal*_ (○, □) and acetate (●, □) over time in experiments testing NO_2_^–^ reduction coupled to Fe(II) oxidation in *Acidovorax* sp. strain BoFeN1 **(A)** and *Paracoccus denitrificans* strain ATCC 19367 **(B)**. Note different time scales. Error bars represent standard error calculated from biological replicates [*n* = 4 **(A)**, 3 **(B)**].

Mixotrophic experiments using strains BoFeN1 and the canonical denitrifier strain ATCC 19367 were set up at equimolar concentrations of NO_2_^–^ and Fe(II) (0.2 mM) and in the presence of acetate only (i.e., no succinate). We investigated if Fe(II) oxidation is somehow enzymatically mediated, and therefore might lead to differences not only in rates of substrate consumption but also in the N and O isotopic fractionation dynamics. Both BoFeN1 (Figure 6A) and strain ATCC 19367 (Figure 6B) were able to fully reduce NO_2_^–^ and oxidize acetate in the presence of Fe(II). Fe(II)_total_ concentrations in both experiments fluctuated significantly and were generally very low (6–20 μM, near LOD). Mineral precipitation was observed immediately after Fe(II)Cl_2_ addition (see Supplementary Figure 1). However, we assume that a large fraction of the Fe(II) added must have been rapidly oxidized at the initiation of the experiment, possibly by traces of O_2_ (Figures 6A,B). Otherwise, Fe(II) concentrations may have been affected by the high sorption affinity of Fe species, resulting in the adsorption of Fe(II) onto the glass wall and thus in the low [Fe(II)] values observed. In general, Fe(II) concentrations are very low and may be additionally biased by the detection limit of the ferrozine assay. Nevertheless, growth of the canonical denitrifier strain ATCC 19367 appeared to be neither enhanced nor impeded by the presence of Fe(II). Contrarily, strain BoFeN1 required more time to reduce NO_2_^–^ and oxidize the organic acid, implying that the presence of Fe(II) somehow affected its growth in general. N_2_O concentrations in the Fe(II)-amended experiments were not quantified.

**FIGURE 7:**
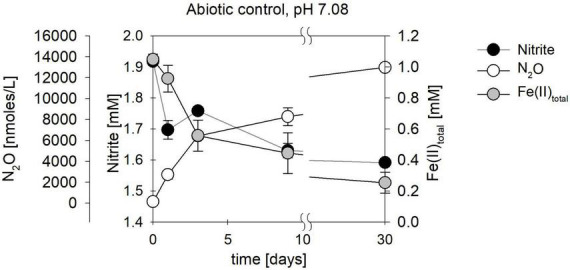
Abiotic nitrite reduction coupled to Fe(II) oxidation at pH 7.08. Nitrite (●) and 1094Fe(II)_total_ (●) concentrations decrease over time, while N_2_O (○) increases. Error bars represent standard error calculated from biological replicates (*n* = 9).

In addition, biological experiments were compared to a purely abiotic control conducted at equimolar concentrations of NO_2_^–^ and Fe(II) (2 mM each). NO_2_^–^ concentrations decreased mainly during the first 10 days of the experiment, reaching a final NO_2_^–^ concentration of 0.33 mM (Figure 7 and Table 3). Concomitantly with NO_2_^–^ reduction, 0.8 mM Fe(II) were oxidized, while roughly 13 μM N_2_O (total) were produced (Figure 7 and Table 3). The reaction was probably catalyzed by the presence of the Fe(II) precipitates formed when Fe(II)Cl_2_ was added to the 22 mM bicarbonate-buffered medium (see Visser et al., 2020). Overall, substrate consumption during enzymatically mediated NO_2_^–^ reduction appeared to be faster compared to the purely abiotic control (Table 3).

**TABLE 3:** Nitrite-based experiments: Concentration changes during heterotrophic growth for all four strains, as well as abiotic and biotic controls (BoFeN1 and the denitrifier 19367 grown under mixotrophic conditions with acetate only); – for reduction, + for production.

Strain	Org. substrate	[mM]	NO_2_^–^[mM]	N_2_O [μM]*	Fe(II) [mM]
*Acidovorax* sp. strain BoFeN1	Acetate	–0.16 ± 0.006	–0.21 ± 0.004	+0.26 ± 0.02	0
	Succinate	–0.16 ± 0.01	–0.21 ± 0.004	+0.09 ± 0.004	0
*Acidovorax delafieldii* strain 2AN	Acetate	–0.22 ± 0.02	–0.18 ± 0.007	+0.61 ± 0.03	0
	Succinate	–0.13 ± 0.02	–0.20 ± 0.0001	+0.22 ± 0.01	0
*“Pseudogulbenkiania ferrooxidans”* strain 2002	Acetate	–0.22 ± 0.02	–0.19 ± 0.002	+0.06 ± 0.008	0
	Succinate	–0.16 ± 0.01	–0.21 ± 0.001	+0.07 ± 0.002	0
*Paracoccus denitrificans* ATCC 19367	Acetate	–0.26 ± 0.02	–0.2 ± 0.0004	+0.06 ± 0.003	0
	Succinate	–0.1 ± 0.005	–0.2 ± 0.0002	+0.09 ± 0.005	0
**Controls**					
Abiotic (2 mM)	Acetate	± 0	–0.33 ± 0.2	+12.9 ± 0.3	–0.79 ± 2.8 × 10^–5^
*Acidovorax* sp. strain BoFeN1	Acetate	–0.06 ± 0.002	–0.18 ± 0.005	–	^#^
*Paracoccus denitrificans* ATCC 19367	Acetate	–0.08 ± 0.004	–0.2 ± 0.006	–	^#^

Values are given as mean concentration consumed or produced ± standard error. Consumption values are calculated by${\bar{\text{X}}}_{\mathrm{t0}}-{\bar{\text{X}}}_{t\,e\,n\,d}$ whereas the production values reflect the maximum concentration produced$({\bar{\text{X}}}_{t,h\,i\,g\,h\,e\,s\,t}-{\bar{\text{X}}}_{\mathrm{t0}}$) and are marked with *.

^#^Fe(II) oxidation in the biotic controls occurred immediately after NO_2_^–^ addition (up to 80%).

#### N and O isotope fractionation during nitrite reduction

**FIGURE 8:**
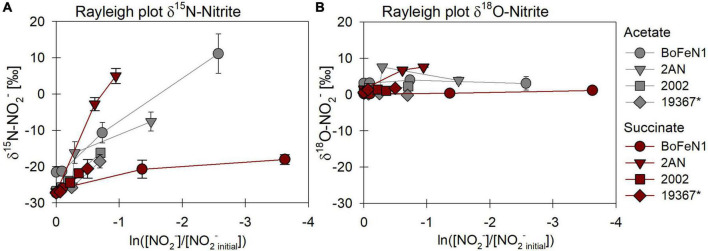
Rayleigh plots of δ^15^N **(A)** and δ^18^O-NO_2_^–^ **(B)** for different bacterial strains grown under heterotrophic conditions [acetate (gray), succinate (red)]. Error bars represent standard error calculated from biological replicates (see Table 4). * Marks the control denitrifier *Paracoccus denitrificans* strain ATCC 19367.

**FIGURE 9:**
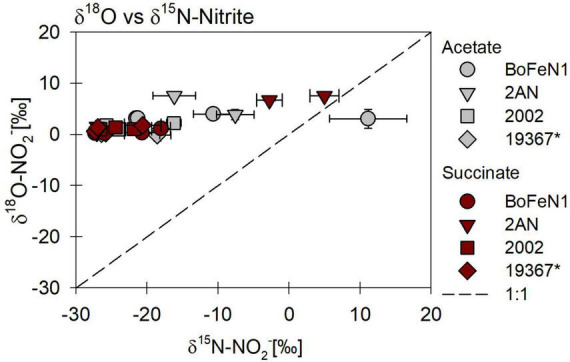
δ^18^O vs. δ^15^N-NO_2_^–^ plot for all four strains grown under heterotrophic conditions, on NO_2_^–^ and in the presence of either acetate or succinate. * Marks the control denitrifier *Paracoccus denitrificans* strain ATCC 19367.

The carbon source did not have a systematic impact on N and O isotope fractionation during nitrite reduction in our experiments (Figure 8). In addition, no systematic differences were observed for isotope effects of NO_2_^–^ reduction by denitrifying vs. putative NDFeOx bacteria (Figure 8 and Table 4). In fact, for NO_2_^–^ δ^18^O in all experiments, the isotope effect was close to zero (data not shown), reflecting a rapid and complete O-atom exchange between NO_2_^–^ and the water in the medium (Figures 8B, 9).

**TABLE 4:** Fractionation factors and *p*-values calculated for the different strains grown on NO_2_^–^ and either acetate or succinate (i.e., without Fe(II)).

Strain	Org. substrate	^15^ε [‰]	*p*-value	*R* ^2^	n
*Acidovorax* sp. strain BoFeN1	Acetate	12.8 ± 1.3	0.3	0.99	4
	Succinate	2.4 ± 1.8	0.005	0.89	4
*Acidovorax delafieldii* strain 2AN	Acetate	12.0 ± 4.7	0.03	0.82	4
	Succinate	34.6 ± 2.9	0.5	0.98	3
*“Pseudogulbenkiania ferrooxidans”* strain 2002	Acetate	15.1 ± 0.1	0.003	0.99	4
	Succinate	12.1 ± 0.7	0.003	0.96	3
*Paracoccus denitrificans* ATCC 19367	Acetate	12.5 ± 1.0	0.002	0.96	4
	Succinate	13.8 ± 0.4	0.009	0.99	3
**Controls (+Fe(II))**					
Abiotic	Acetate	–12.4 ± 1.3	6.2 × 10^–6^	0.40	5
*Acidovorax* sp. strain BoFeN1	Acetate	17.4 ± 2.4	0.03	0.95	4
*Paracoccus denitrificans* ATCC 19367	Acetate	14.7 ± 0.1	0.13	0.99	3

Red—p-value > 0.05, ^18^ε values are not shown since they are clearly impacted by O atom exchange with the medium.

Controls include an abiotic experiment [2 mM Fe(II)/2 mM NO_2_^–^; pH 7.1] and two strains grown on NO_2_^–^, Fe(II) and acetate. ± Standard error. Since the decrease in NO_2_^–^ during the abiotic experiment was non-systematic, not following Rayleigh model dynamics, the calculated Rayleigh isotope effect is not realistic and thus is given in red.

**FIGURE 10:**
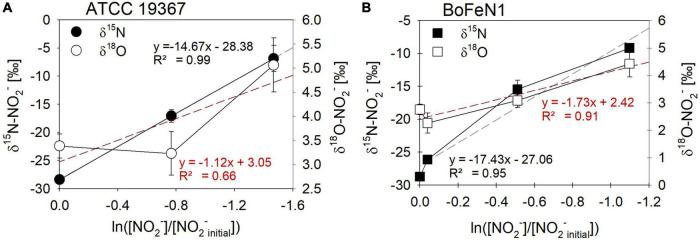
Rayleigh plots for δ^15^N- (●, □) and δ^18^O- (○, □) NO_2_^–^ values for the control denitrifier *Paracoccus denitrificans* strain ATCC 19367 **(A)** and *Acidovorax* sp. strain BoFeN1 **(B)** grown mixotrophically in the presence of Fe(II) and acetate. Equations and dotted lines in black represent the linear regressions for δ^15^N, whereas red lines represent δ^18^O. Note that for δ values, different scales apply. Error bars represent standard error calculated from biological replicates [*n* = 4 **(A)**, 3 **(B)**].

For *Acidovorax* sp. strain BoFeN1 and the canonical denitrifier strain ATCC 19367, NO_2_^–^ N and O isotope effects were determined under mixotrophic conditions with equimolar concentrations of NO_2_^–^ and acetate, and at low concentrations of Fe(II) (Figure 10; see concentration trends above). Both, δ^15^N-NO_2_^–^ and ^15^ε values were similar to those measured under heterotrophic conditions (Figure 10 and Table 4). Again, the apparent O-isotope fractionation during NO_2_^–^ reduction was very low (Figure 10). Except for BoFeN1 grown on succinate, most ^15^ε-NO_2_^–^ values varied between 12 and a maximum of 15‰. Although overall consumption patterns were comparable to the other strains (Figure 5 and Table 3), strain 2AN on succinate yielded the highest ^15^ε value (∼34‰). The corresponding high *p*-value (0.5), however, indicates that the reaction observed does not follow the rules of a classical Rayleigh distillation, and possibly indicates that multiple processes might be at work. Contrarily, in strain BoFeN1, succinate-amendment resulted in a very low ^15^ε-NO_2_^–^ value (2.4‰, *p*-value = 0.005, *R*^2^ = 0.89), suggesting that here the availability of succinate dramatically decreased the expression of ^15^ε.

**FIGURE 11:**
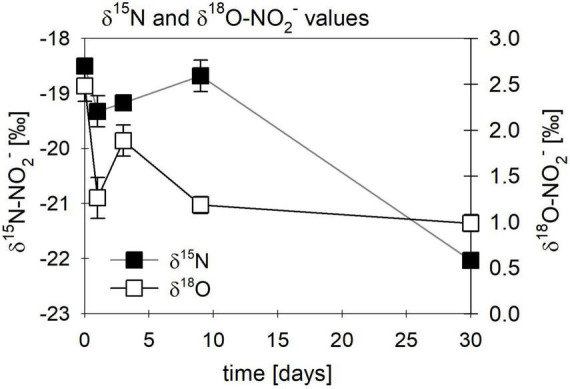
Changes in δ^15^N (■)/ δ^18^O-NO_2_^–^ (□) values observed in abiotic nitrite reduction coupled to Fe(II) oxidation over time. Error bars represent standard deviation (*n* = 3).

While the biotic controls that were grown mixotrophically exhibited isotope effects similar to those observed for heterotrophic NO_2_^–^ reduction, the purely abiotic experiment (even though isotope effects were not calculated) revealed isotopic trends that were clearly distinguishable from our biotic experiments (compare Figure 11, Supplementary Figure 2 and Table 4). Under purely abiotic conditions, δ^15^N and δ^18^O-NO_2_^–^ values seem to have decreased over time (Figure 11), following a transient inverse trend. However, δ^18^O-NO_2_^–^ values are relatively low and most likely do not represent the kinetic isotope effect but, again, the rapid O-atom exchange between NO_2_^–^ and ambient water.

## Discussion

### Factors controlling N and O isotope fractionation during heterotrophic NO_3_^–^ reduction

The observed changes in δ^15^N- vs. δ^18^O-NO_3_^–^ values are consistent with previously reported findings and support that N and O isotope fractionation during heterotrophic denitrification is mainly regulated by the mechanism binding NO_3_^–^ to the enzyme reactive site (Granger et al., 2008; Treibergs and Granger, 2017; Asamoto et al., 2021). Furthermore, the pooled average ^15^ε values (hereafter, ^15^ε_total_) calculated for heterotrophic NO_3_^–^ reduction (17.6 ± 2.8‰) fall well within the range (^15^ε-NO_3_^–^: 15–25‰) for Nar-mediated NO_3_^–^ reduction (Granger et al., 2008; Karsh et al., 2014; Chen et al., 2020). Although minor variations in N and O isotope fractionation were observed, the direct comparison between the organic acid treatments (i.e., acetate vs. succinate) based on the maximum consumption (see Table 1) calculated for each setup (heterotrophic vs. mixotrophic) revealed similar trends, further supporting that an alteration of the organic acid does not systematically impact overall reaction dynamics.

When distinguishing Nap- vs. Nar-driven nitrate isotope fractionation, values of ^18^ε:^15^ε reported for these nitrate reductases are known to serve as reliable tool since they are considered to result from different binding affinities and reducing capacities of the respective enzyme (Granger et al., 2008; Karsh et al., 2012; Granger and Wankel, 2016). The ^18^ε:^15^ε value (1.1 ± 0.2‰) calculated for all strains grown under heterotrophic conditions is similar to values previously reported (close to 1) for Nar-driven fractionation (e.g., Granger et al., 2008; Asamoto et al., 2021). Yet, the overall average is probably slightly biased due to a rather high ^18^ε:^15^ε value obtained for the canonical denitrifier *P. denitrificans* strain ATCC 19367 on acetate (∼2.5). Although organic acid consumption and NO_3–_ reduction were complete (organic acid: –0.63 ± 0.007 mM, NO^3–^: –0.76 ± 0.11 mM), and similar to the averaged values calculated for the other setups (organic acid: –0.52 ± 0.03 mM, NO^3–^: –0.73 ± 0.03 mM), N isotope fractionation in strain ATCC 19367grown on acetate was much lower (^15^ε: 8.4 ± 0.5‰ vs. ^15^ε_total_: 17.6 ± 2.8‰). Yet, O isotope fractionation patterns were comparable to the other setups (^18^ε: 21.2 ± 2.7‰ vs. ^18^ε_total_: 18.1 ± 2.5‰), and therefore yield a higher ^18^ε:^15^ε value. Excluding strain ATTC 19367 on acetate from the calculation results in a total ^18^ε:^15^ε value that is slightly lower (0.94 ± 0.01‰), but still falls directly within range reported for Nar-driven N and O isotope fractionation (Granger et al., 2008; Granger and Wankel, 2016).

Granger et al. (2008) previously emphasized the relevance of denitrification-related enzymes in ordaining N and O isotope fractionation (e.g., different isotope effects for Nar vs. Nap). Yet other studies have argued that physico-chemical factors, including environmental conditions (Zumft, 1997; Kritee et al., 2012) and cellular uptake mechanisms involved, as well as specific nitrate reduction rates (Needoba et al., 2004; Kritee et al., 2012; Wunderlich et al., 2012; Karsh et al., 2014; Denk et al., 2017), might modulate the enzyme-level isotope partitioning. For example, factors such as the initial NO_3_^–^ concentrations, turbulence during growth, C_org_ and nutrient conditions in general, the growth phase of the transferred batch culture, and of course the presence of O_2_, have been suggested to potentially impact expression of N and O isotope fractionation during denitrification, and thus alter apparent ^15^ε and ^18^ε values (Granger et al., 2008; Kritee et al., 2012). Since all strains were cultivated under the exact same growth conditions (anoxic, no shaking, same medium/temperature, dark), cultivation conditions as a factor contributing to variations detected in N and O isotope fractionation can be excluded. Again, an alteration of the organic carbon substrate did not seem to affect the NO_3_^–^ N and O isotope fractionation in any systematic way in our experiments, and the minor variations discerned in isotopic trends appeared to be mostly strain-related. Both ^15^ε and ^18^ε values between acetate- (^15^ε: 22.3 ± 3.1‰; ^18^ε: 21.2 ± 2.3‰) and succinate- (^15^ε: 21.2 ± 1.0‰; ^18^ε: 20.0 ± 1.3‰) amended setups were quite similar.

The minor variances probably indicate that the nature of the carbon source may partly regulate expression of ^15^ε in these strains. Differences in observed ^15^ε values between other studies and those reported here for *P. denitrificans* strain ATCC 19367 may be linked to the different cultivation conditions, including generally lower concentrations tested here (see e.g., Granger et al., 2008; Kritee et al., 2012). Interestingly, strain 2002 grown on acetate was the only setup showing slightly lower organic acid consumption (–0.29 ± 0.04 mM) compared to the other setups (total averaged: –0.54 ± 0.23 mM), which could possibly have resulted in lower ^15^ε and ^18^ε values and thus would support that organic acid reaction dynamics are coupled to Nar activity. Still, the ^18^ε:^15^ε value (0.92) of strain 2002 on acetate supports Nar-driven fractionation. Considering ^18^ε-NO_3_^–^ values, a similar magnitude was observed for both *Acidovorax* strains, ranging from 18.9 to 23.6‰ and 17.2 to 18.7‰ for acetate and succinate, respectively, whereas the ^18^ε-NO_3_^–^ value for strain 2002 grown on acetate was much lower (2.7 ± 0.8‰) compared to ^18^ε-NO_3_^–^ value from succinate-amended batches (20.0 ± 1.3‰). In contrast to what was observed for the N isotope effect, ^18^ε-NO_3_^–^ values for strain ATCC 19367 did not significantly vary between acetate and succinate amendment, ranging from 21.2 to 22.1‰. The equivalent values obtained for the strain ATCC 19367 suggest that the lower ^15^ε-NO_3_^–^ values detected are not necessarily based on genetic variations between the strains. A clear modulating role of the type of substrate concerning the apparent difference in isotope fractionation for the strains tested is not indicated. Whether these low values are strain-specific or even originate from enzymatic differences (nitrate reductase subunits, binding mechanisms at the enzyme’s reactive site) (Asamoto et al., 2021), however, remains unclear.

Previous studies suggested that N and O isotope effects depend on not only Nar-induced fractionation dynamics, but also on the overall reaction kinetics, and thus are linked to substrate availability. For example, Wunderlich et al. (2012) showed that the change from a short-chained organic acid (e.g., acetate) to a more complex organic (e.g., benzoate) substrate can influence N and O isotope fractionation during heterotrophic denitrification. They attributed the organic substrate-dependent difference in fractionation to changes in the relative kinetics of NO_3_^–^ transport (uptake) compared to the kinetics related to NO_3_^–^ reduction within the cell (cell-specific reduction rate) (Wunderlich et al., 2012). Microorganisms are known to adjust their catabolic pathways according to the carbon source available (Ornston, 1971; González-Cabaleiro et al., 2015), yet electron transfer during dissimilatory NO_3_^–^ respiration follows the same direction along the electron transport chain (Ornston, 1971; Wunderlich et al., 2012). Thus, by defining, which catabolic pathway is utilized, the electron donor ultimately impacts the overall reaction kinetics of the electron acceptor (NO_3_^–^) and thus its isotope fractionation (Granger et al., 2008; Wunderlich et al., 2012). This should, however, also be reflected in differential microbial growth patterns, which were not observed here, notwithstanding the succinate-amended setups exhibiting a slightly shorter lag phase (∼5 h). Furthermore, recent work has also provided evidence for the ability of carbon substrates to modulate not only microbial metabolic pathways, but also gene expression, and thus to impact e.g., NO_3_^–^ reduction (Sears et al., 2000; Carlson et al., 2020). Therefore, N and O isotope fractionation could be impacted if expression of Nar/Nap or even transport related enzymes, are indeed regulated by carbon substrate availability, depending on their specific energy yield (González-Cabaleiro et al., 2015). However, Granger et al. (2008, 2010) argued that isotope effects associated with cellular transport are comparatively small, assuming that NO_x_ uptake happens most likely *via* diffusion into the periplasm followed by active transport into the cytoplasm. Conversely, Denk et al. (2017) suggest that a net N isotope effect of ∼5.9 ± 3.7‰ for the uptake of NO_3_^–^ by free-living microorganisms should be considered.

Since it has been shown that, e.g., in *E. coli*, specific proteins such as NarK and NarU facilitate NO_3_^–^/NO_2_^–^ uptake into the periplasm, as well as the export of NO_2_^–^ out of the cytoplasm (Moir and Wood, 2001; Jia et al., 2009), the possible impact on isotope effects associated with an active transport system may need to be reconsidered. This implies that N and O isotope fractionation might not only be controlled by the transporters directly regulating NO_3_^–^ uptake, but also by processes controlling the uptake of the organic acid. Moreover, if indeed the compound-specific properties of the organic acid (or electron donor in general) modulate the expression of transporter and even reductases, then uptake/consumption and the overall dynamics of the electron transport chain could differ, leading to variations in the total reaction kinetics and possibly variations in N and O isotope fractionation patterns. Kritee et al. (2012) showed that ^15^ε_Nar_ appeared to be strongly impacted by the reducing power of the carbon source under NO_3_^–^-replete conditions. Based on an influx/efflux model, wherein NO_3_^–^ uptake rates strongly depend on the energy yields produced during respiratory NO_3_^–^ reduction and result in an increased sensitivity of uptake toward changes in NO_3_^–^ reduction rates, they concluded that NO_3_^–^ uptake heavily regulates isotope fractionation dynamics (Kritee et al., 2012). Following their hypothesis that NO_3_^–^ uptake is governed by nutrient/energy availability, low levels of nutrients and/or substrates having low energy yields might act to decrease NO_3_^–^ uptake and result in lower ^15^ε values (Kritee et al., 2012). Hence, the high ^18^ε:^15^ε (∼2.5) value measured in strain ATCC 19367 on acetate might even be interpreted as reflecting some sort of nitrate transport-related isotope discrimination.

Considering that microbes need to utilize different carrier systems or transport mechanisms for different organic substrates (electron donors), which would then regulate the coupled uptake of the electron acceptor, an impact on the overall reaction kinetics seems plausible (e.g., Bosdriesz et al., 2015). Hence, different uptake mechanisms during denitrification could therefore result in different N and O isotope fractionation dynamics (Wunderlich et al., 2012; Karsh et al., 2014; Martin and Casciotti, 2016). Again, the reduced lag phase (∼5 h) observed in succinate-amended batch cultures might indicate an apparent enhancement in growth, possibly also supporting the presence of an active, and thus more efficient, cellular uptake mechanism (Groeneveld et al., 2010; Valentini et al., 2011). Particularly, in the case of heterotrophic growth, the uptake of organic acids is generally regulated actively by substrate-specific transporter proteins, which are located within the cell membrane (Gutowski and Rosenberg, 1975; Jolkver et al., 2009). For example, the cellular uptake of succinate is known to be mediated by carriers of TRAP-transporters (Groeneveld et al., 2010; Valentini et al., 2011; Unden et al., 2016), which are, according to the published genome sequences (Supplementary Section 1.3), present in all strains tested here. Hence, succinate amendment and thus corresponding TRAP transporter activation might indeed have influenced overall reaction dynamics within the cell, thereby enhancing growth. Conversely, for the uptake of acetate, diffusion has been proposed as main uptake mechanism for cellular uptake (Jolkver et al., 2009). Nevertheless, consistent with our results, the differences between toluene (^15^ε-NO_3_^–^: 18.1–7.3‰; ^18^ε-NO_3_^–^: 16.5–16.1‰), benzoate (^15^ε-NO_3_^–^: 18.9 ‰; ^18^ε-NO_3_^–^: 15.9‰) and acetate-related (^15^ε-NO_3_^–^: 23.5–22.1‰; ^18^ε-NO_3_^–^: 23.7–19.9‰) N and O isotope fractionation reported by Wunderlich et al. (2012), were, overall, rather subtle, with considerable overlap between isotope-effect ranges for different substrates. This may support that the reductase is indeed the sole origin for the isotope effects observed, and that, if at all, the organic substrate type plays a subordinate role in modulating the N and O isotope effect during heterotrophic denitrification. While more complex (i.e., cyclic) organic acids might increase N and O isotope fractionation, our results suggest that the type of short-chain organic acids tested here did not consistently influence NO_3_^–^ N and O isotope effects. This implies that the uptake mechanisms utilized for the tested organic acids are either the same, or simply do not translate into physiological variations in nitrate-related reaction kinetics.

However, the slight variations in N and O isotope fractionation observed might possibly originate from NO_2_^–^, which is the first intermediate product in the denitrification pathway. In general, we did not observe NO_2_^–^ accumulation and, except for strain 2002 on acetate (+0.53 ± 0.1 mM) and the canonical denitrifier strain ATCC 19367 (acetate: +0.18 ± 0.02 mM succinate: +0.28 ± 0.11 mM), maximum NO_2_^–^ levels remained low. Yet, nitrite-water exchange, incomplete NO_2_^–^ efflux or NO_2_^–^ back-reaction, could still have influenced reaction kinetics. Considering that particularly in acetate-amended setups of strain 2002 and the strain ATCC 19367 lower ^15^ε values were observed, a link between N isotope fractionation and [NO_2_^–^] and/or NO_2_^–^ production/consumption dynamics cannot be excluded. However, recently published results from ^18^O tracer experiments showed that neither a back-reaction nor an O-atom exchange between nitrite and water takes place during denitrification (Asamoto et al., 2021). Incomplete NO_2_^–^ efflux, on the other hand, might still play an important role in denitrification reaction dynamics. Isotope tracer experiments conducted by Jia et al. (2009), investigating NO_3_^–^ and NO_2_^–^ uptake mechanisms in *E. coli*, revealed a mechanistic linkage between NO_3_^–^ uptake and reduction to NO_2_^–^ expulsion. From their experiments, they concluded that NarK and NarU act as nitrate-nitrite antiporters, implying that an export of NO_2_^–^ into the periplasm is required before it can be further reduced in the denitrification pathway (Jia et al., 2009). Whether the export into the peri-/cytoplasm and/or the partial (extracellular) accumulation of NO_2_^–^ does indeed result in variations in the expression of isotope effects during NO_3_^–^/NO_2_^–^ reduction, remains unclear.

Overall, the observed enhancement in growth (i.e., the reduced lag phase), although not directly affecting N and O isotope fractionation, might simply be related to the metabolic pathway of organic acid utilization *via* anaerobic respiration. Here, the consumption of acetate might be coupled to the formation of acetyl-CoA *via* a succinyl-CoA:acetate-CoA transferase, which transfers the organic acid in the citric acid cycle (Galushko and Schink, 2000; Yoon et al., 2013). In contrast, succinate utilization is immediate since it is an intermediate of the Krebs cycle. Hence, it can be transferred, and used directly, in the last steps of the Krebs cycle, which could explain the slightly shorter lag phase observed in succinate-amended experiments (Thauer, 1988; Groeneveld et al., 2010).

### Factors controlling N and O isotope fractionation during heterotrophic NO_2_^–^ reduction

The ^15^ε_total_ value for ^15^N-NO_2_^–^ of 14.4 ± 3.2‰ is slightly higher compared to previously reported NirS-driven N and O isotope fractionation, in which average calculated values were 8 ± 2‰ (Martin and Casciotti, 2016). Our results thus imply a roughly 6‰ higher N isotope effect compared to previous reports on ^15^ε_NirS_ values, but are still lower than the range reported for NirK-driven NO_2_^–^ reduction (20–26‰) (Martin and Casciotti, 2016). Although not directly applicable, previous arguments based on the efflux model (Kritee et al., 2012) could serve as a plausible basis for the observed differences. Assuming that specific proteins such as NarK and NarU facilitate NO_3_^–^/NO_2_^–^ uptake into the periplasm, as well as the export of NO_2_^–^ out of the cytoplasm (Moir and Wood, 2001; Jia et al., 2009), NO_2_^–^ uptake might also be nutrient/energy limited (Kritee et al., 2012). Hence, mechanisms involved in NO_2_^–^ uptake/efflux, either directly (i.e., by active NO_2_^–^ transport *via* membrane proteins) or indirectly (i.e., by modulating gene expression or providing sufficient energy levels to sustain the electron transport chain), could possibly impact reaction kinetics and isotope fractionation during NO_2_^–^ reduction. In addition, previous studies emphasized the role of culture conditions on the expression of isotope effects during nitrate (Granger et al., 2008; Kritee et al., 2012) and nitrite (Bryan et al., 1983; Martin and Casciotti, 2016) reduction. In contrast to our study, Martin and Casciotti (2016) grew cultures on a nutrient-rich medium (Tryptic Soy broth) at room temperature under non-strict anoxic conditions and constant shaking. Although NO_2_^–^ concentrations were lower (0.1 mM) relative to our study (0.2 mM), the high nutrient supply from the medium would enhance overall growth performance, whereas disturbance (shaking) and lower temperatures would presumably have the opposite effect. For NO_3_^–^ reduction, Kritee et al. (2012) argued that decreasing isotope effects are linked to decreasing substrate concentrations and cell-specific reduction rates. In addition, Bryan et al. (1983) observed that an increase in NO_2_^–^ concentrations resulted in increasing N isotope effects for NO_2_^–^ reduction. However, a 6‰ increase in the ^15^ε_NirS_ value solely resulting from doubling the NO_2_^–^ concentration (0.2 vs. 0.1 mM) appears questionable.

Transport mechanisms and thus reaction kinetics for NO_2_^–^ differ from NO_3_^–^ reduction, which is reflected for example by an up to 10-fold more active NO_2_^–^ uptake reported for NirC (Jia et al., 2009), a protein known for catalyzing NO_2_^–^ transport across the membrane (Jia et al., 2009; Lü et al., 2012). Considering the potential toxicity of NO_2_^–^ (Bollag and Henninger, 1978; Van Cleemput and Samater, 1995; Lü et al., 2012; Yoon et al., 2015), a more dynamic NO_2_^–^ uptake and efflux by cells (Bryan et al., 1983) is plausible. Hence, concentrations near a cellular toxicity threshold could result in extracellular NO_2_^–^ accumulation and thus affect reaction kinetics. Here we assume that the NO_2_^–^ toxicity effect is negligible since the NO_2_^–^ experiments were conducted at much lower concentrations (0.2 mM, 0.1 Mm; Martin and Casciotti, 2016), compared to previously published NDFeO studies, in which maximal NO_2_^–^ accumulation for strain BoFeN1 of ∼2 mM NO_2_^–^ was reported (Klueglein and Kappler, 2013; Klueglein et al., 2014). This is also supported by the fact that, under heterotrophic conditions, most strains did not exhibit NO_2_^–^ accumulation. Considering that NirC expression in e.g., *E. coli* has been shown to be induced by certain transcription factors, i.e., NarP, NarL, and FNR (anoxic conditions) (Lü et al., 2012), the observed differences in isotopic fractionation could be related to the fact that cultures in our experiments were grown under strictly anoxic conditions without any initial nitrate supply. This initial availability of O_2_ and NO_3_^–^ reported by Martin and Casciotti (2016) could have resulted in a reduced and increased transcription of FNR and NarL, respectively, which in turn influenced the expression of NirC. During NO_3_^–^ reduction, previous studies have shown that a higher cell-specific activity and thus higher reduction rates result in higher ^15^ε values (Kritee et al., 2012; Karsh et al., 2014). Hence, the higher ^15^ε values observed here could result from the cultivation conditions applied, possibly leading to an increased expression of NirC and thus to faster transport kinetics, which could thereby enhance cell-specific activity and thus reduction rates. Future studies should address these dynamics.

As described above for NO_3_^–^ reduction, the type of carbon substrate could also affect NO_2_^–^ N and O isotope effects. Substrate availability and correspondingly attainable energy yields might modulate transport mechanisms involved in NO_2_^–^ uptake and efflux, influencing the electron transport chain and, thus, overall reaction kinetics. In general, however, growth on each of the two carbon sources did not reveal any distinct impact on overall NO_2_^–^ N and O isotope fractionation. Only the two *Acidovorax* strains deviate from the other strains with regards to N isotope fractionation when grown on succinate. However, whereas availability of succinate to strain BoFeN1 led to a decreased expression of ^15^ε, succinate-amendment appears to have increased ^15^ε values in strain 2AN. For the latter, however, the poor linear fit to Rayleigh dynamics suggests that the reaction may have been influenced by multiple processes. This might be further supported by the steep decreases observed in NO_2_^–^ and succinate concentrations (Figures 5D,E), as well as by the fast and steep increase in N_2_O, also resulting in the highest levels observed (+0.61 ± 0.03 μM) (Figure 5F). These observations possibly support that, in addition to Nir-dependent expression, the overall isotope effect here could arise as the result of a complex multistep process (e.g., Granger and Wankel, 2016). Again, the lag phase in the succinate-amended experiments was slightly shorter (∼5 h), which points to a more immediate utilization of succinate as an intermediate in the Krebs cycle (Thauer, 1988; Groeneveld et al., 2010). In contrast, acetate must be converted to acetyl-CoA before it can be shunted into the citric acid cycle (Galushko and Schink, 2000; Yoon et al., 2013), which may explain the enhanced growth in succinate-amended cultures.

In the NO_2_^–^ experiments, ^18^ε-values were rather low (0–1‰), except for strain 2AN on acetate (14.6 ± 3.9‰) and succinate (6.7 ± 1.1‰), which would fall within the range reported for NirS-driven O isotope fractionation (Martin and Casciotti, 2016). However, as depicted in Figure 9, ^18^ε-values are most likely the result of rapid O isotope equilibration with the medium, as noted previously (Martin and Casciotti, 2016).

### Iron and its possible impacts on N and O isotope fractionation during mixotrophic NO_x_ reduction

Values of ^15^ε-NO_3_^–^ (17.5–24.3‰) and ^18^ε-NO_3_^–^ (16–23.4‰) obtained under mixotrophic growth conditions, containing Fe(II), showed no significant differences compared to the heterotrophically-grown experiments (see above) and thus also fall within range reported for classical denitrification (e.g., Granger et al., 2008). Yet, considering the proposed direct enzymatic coupling between Fe(II) oxidation and nitrate reduction, N and O isotope fractionation dynamics should elucidate the mechanistic details of this reaction, especially, since the presence of Fe(II) in denitrifying microbial communities has been reported to impact microbial enzymatic activity and thus microbial physiology (Laufer et al., 2016; Zhou et al., 2016; Nordhoff et al., 2017; Tominski et al., 2018b; Tian et al., 2020). Hence, if these NDFeOx bacteria indeed switch from chemoorganotrophic (NO_x_ + C_org_) to chemolithotrophic (NO_x_ + Fe(II)) denitrification, this should affect reaction kinetics and, thus, should be reflected in the isotope values.

Here, we compared mixotrophic and heterotrophic NO_3_^–^ reduction, yet no differences in either growth, substrate consumption nor N and O isotope fractionation were discernible. Since true chemolithoautotrophic Fe(II) oxidation is known be limited by the available amount of energy due to a low redox differential between nitrate and e.g., FeCO_3_/Fe(OH)_3_, lower denitrification rates compared to chemoorganotrophic denitrification can be expected (Devlin et al., 2000; Di Capua et al., 2019). In turn, chemoorganotrophic denitrification commonly results in a faster nitrate consumption (e.g., Chen et al., 2020). If these strains utilize indeed both processes in the mixotrophic setups, either simultaneously or consecutively, the different dynamics in reaction kinetics are likely reflected in a distinguishable N and O isotope systematic (see Supplementary Figure 3). However, no differences between mixotrophic and heterotrophic growth condition were discernible with regards to the isotopic pattern in association with substrate consumption. The fact that Fe(II) oxidation and organic acid consumption occurred simultaneously suggests that the processes may indeed be coupled, but NO_3_^–^ reduction may nevertheless not be linked to a purely enzymatic Fe(II) oxidation. Our data indicate that the putative NDFeOx bacteria tested here do either not possess an specific enzymatic system for Fe(II) oxidation or that, under the conditions tested here, a switch from chemoorganotrophic to chemolithotrophic denitrification is simply not favorable.

Furthermore, other processes possibly affecting cellular uptake (i.e., cell encrustation) can also be excluded, since, in contrast to previous studies, concentrations tested here were much lower (<1 mM). Cell encrustation has been observed in various studies investigating NDFeO at high NO_3_^–^ and Fe(II) concentrations (>2 mM) (Kappler et al., 2005; Schädler et al., 2009; Klueglein et al., 2014; Liu et al., 2018; Chen et al., 2020). During NDFeO, the precipitation of highly redox-reactive Fe(III) (oxyhydr)oxides has been reported to occur within (cyto-/periplasm), as well as at the cell surface (e.g., Schädler et al., 2009). Their presence might not only promote additional reactions (Coby and Picardal, 2005; Visser et al., 2020), but also block cellular uptake mechanisms and thus has been proposed to alter mass transfer processes prior to N-O bond cleavage, which in turn could impact isotope fractionation dynamics (Chen et al., 2020). However, growth performance in mixotrophic experiments was not reduced relative to cultures grown under heterotrophic conditions, suggesting that, at these low concentrations, microbial cells were not impacted by Fe(III) (oxyhydr)oxide precipitation. Furthermore, Chen et al. (2020) observed similar ^15^ε and ^18^ε –NO_3_^–^ values for strain 2002 grown in the presence (^15^ε: 24.1 ± 2.4‰; ^18^ε: 16.8 ± 3.4‰) and absence (^15^ε: 25.1 ± 2.4‰, ^18^ε: 12.2 ± 2.1‰) of Fe(II). These values are comparable to our results of strain 2002, although our ^15^ε (^∼^23‰) and ^18^ε values (∼22‰) are almost equal. In contrast to our results, their ^18^ε:^15^ε values were much lower (+Fe(II): 0.73 ± 0.13, –Fe(II): 0.50 ± 0.08), which they attribute to a Nap-induced NO_3_^–^ reduction (Chen et al., 2020). Chen et al. (2020) argued that the expression of Nar possibly depends on growth conditions (i.e., presence of vitamins, salts), which in their case favored the expression of the *napA*-encoding gene present in the genome of strain 2002 (Byrne-Bailey et al., 2012) and thus promoted NO_3_^–^ reduction *via* Nap. They also suggested that both reductases, i.e., Nar and Nap, are present, however, Nar is less active during NDFeO (Chen et al., 2020). In our experiments, concentrations were much lower and mixotrophic growth of strain 2002 was only promoted in the presence of succinate, resulting in ^18^ε:^15^ε value of ∼1 and thus supporting a Nar-driven NO_3_^–^ reduction instead. Furthermore, the differences in ^18^ε –NO_3_^–^ values observed between mixotrophic and heterotrophic growth, which are apparently attributed to the influence of Fe(II) on isotope fractionation (Chen et al., 2020), are not supported by our findings and thus do not appear to hold at lower substrate concentrations. Nevertheless, mixotrophic and heterotrophic growth conditions in both studies did not reveal clear differences in N isotope fractionation dynamics and thus indicate that Fe(II) oxidation does not seem to be directly linked to NO_3_^–^ reduction (Chen et al., 2020). One potential explanation for the observed oxidation of Fe(II) is based on previous findings, involving *c*-type cytochromes that modulate the electron transfer from Fe(II) to the cellular membrane (Liu et al., 2012; Ishii et al., 2016). Liu et al. (2018) showed that, *c*-type cytochromes within the extrapolymeric substances (EPS) excreted by strains such as BoFeN1, could promote Fe(II) oxidation, implying that this process is not directly facilitated by their cellular metabolism. Hence, considering these findings and our results, an enzymatically-mediated pathway for Fe(II) oxidation, albeit not energetically linked to N utilization, cannot be excluded (see Dopffel et al., 2022).

Since growth was not observed for all strains in the presence of Fe(II) (i.e., canonical denitrifier strain ATCC 19367, strain 2002 on acetate), succinate amendment appears to have enhanced growth performance, resulting in reproducible Fe(II) oxidation patterns in the replicates of strains BoFeN1, 2AN and 2002 (Figure 3). Yet, consistent with previous experiments under heterotrophic growth conditions, the type of the organic substrate did not discernibly influence the observed N and O isotope fractionation dynamics. Furthermore, in contrast to other studies utilizing similar media, but higher initial concentrations, no NO_2_^–^ accumulation was observed (e.g., Klueglein and Kappler, 2013; Klueglein et al., 2014) and NO_2_^–^ concentrations decreased toward the end of the experiments (Table 1). In mixotrophic experiments, maximum NO_2_^–^ concentrations were slightly higher and N_2_O production patterns differed notably, yielding overall higher concentrations (Table 1). Considering that chemodenitrification, i.e., the abiotic reaction between Fe(II) and NO_2_^–^, supposedly results in higher N_2_O yields (e.g., Jones et al., 2015), and that so far no evidence has been found supporting an abiotic reaction between Fe(II) and NO_3_^–^ (e.g., Margalef-Marti et al., 2020), our combined results possibly indicate that chemodenitrification, rather than an enzymatic pathway, might be involved in Fe(II) oxidation. Yet, which N-species is actually oxidizing Fe(II) is not really clear (see below).

Due to a potentially enhanced toxicity effect caused by the coupled presence of NO_2_^–^ and Fe(II) (Bollag and Henninger, 1978; Andrews et al., 2003; Lü et al., 2012; Klueglein et al., 2014) mixotrophic NO_2_^–^ reduction was only tested with the canonical denitrifier strain ATCC 19367 and the putative NDFeOx strain BoFeN1 in a control experiment amended with acetate. The N isotope effects were higher for strain BoFeN1 (^15^ε-NO_2_^–^ 17.4 ± 2.4‰) and for strain ATCC 19367 (^15^ε-NO_2_^–^ 14.7 ± 0.1‰), compared to the heterotrophic setups (BoFeN1: ^15^ε-NO_2_^–^ ∼12.8‰, 19367: ^15^ε-NO_2_^–^ ∼12.5‰). In addition, our values obtained for mixotrophic growth are roughly ∼8‰ higher compared to previously published results on NO_2_^–^ reduction by NirS (Martin and Casciotti, 2016). Nevertheless, the presence of Fe(II) did not have a distinct impact on nitrite N isotope fractionation by strain BoFeN1 or strain ATCC 19367, and N isotope fractionation dynamics are similar to what was observed when grown heterotrophically. The slightly higher ^15^ε-NO_2_^–^ values could, however, indicate that isotope fractionation is indeed impacted by slightly enhanced reaction kinetics resulting from a faster uptake by an active NirC (see above), and that NirC-related uptake is somehow enhanced in the presence of Fe(II). Nevertheless, the fact that heterotrophic and mixotrophic NO_2_^–^ reduction resulted in only minor differences in N isotope fractionation patterns does indicate that either N isotope fractionation dynamics are less sensitive with regards to chemodenitrification, or that Fe(II) oxidation is not directly linked to NO_2_^–^ reduction.

Therefore, the abiotic control experiment might help to better understand the dynamics dominating this system. The δ^15^N-NO_2_^–^ measured during the purely abiotic control experiment (w/o cells), which was conducted at higher concentrations and in the presence of highly amorphous Fe(II)_3_(PO_4_)_2_, indicates a transient inverse isotope effect (Figure 11). However, determining a reliable ^15^ε –NO_2_^–^ value is not possible since neither NO_2_^–^ concentrations nor δ^15^N-NO_2_^–^ values did follow classical Rayleigh model dynamics (see Supplementary Figure 2). Nevertheless, neglecting the violation of true Rayleigh requirements, a ^15^ε-NO_2_^–^ value of –12.4 ± 1.3‰ can be approximated. Hence, with regards to overall reaction dynamics (i.e., NO_2_^–^ consumption, N_2_O production, Fe(II) oxidation), but also the apparent N isotopic trends, abiotic Fe(II oxidation coupled to NO_2_^–^ reduction seems to be clearly distinguishable from NO_2_^–^ reduction isotope patterns in experiments with denitrifying bacteria, in presence or absence of Fe(II). This is also supported by a recently published study, which investigated Fe(II) mineral-driven NO_3_^–^ and NO_2_^–^ reduction in polluted groundwater (Margalef-Marti et al., 2020). Although also experiencing slight fluctuations, their testing of the abiotic reaction between 1 mM NO_2_^–^ and 5 mM Fe(II) resulted in ^15^ε-NO_2_^–^ values ranging from –14.1 to –17.8‰ (*R*^2^ > 0.89) (Margalef-Marti et al., 2020), which are somewhat comparable to our results (Supplementary Section 1.2). Furthermore, the abiotic reaction has been shown to be enhanced by the presence of reactive surfaces such as minerals, or simply microbial cell surfaces (Margalef-Marti et al., 2020; Visser et al., 2020), even exhibiting a sizable catalytic effect in the presence of dead organic biomass, resulting in an ^15^ε isotope effect for ^15^N-NO_2_^–^ of 10.3‰ (Visser et al., 2020). Hence, the highly reactive Fe(II) minerals, the presence of which was supported by precipitate formation observed in all mixotrophic experiments, as well as the cell biomass, could additionally enhance Fe(II) oxidation (Grabb et al., 2017; Sheng et al., 2020; Visser et al., 2020).

In light of the abiotic control experimental results our N isotopic data do not support Fe(II) oxidation directly coupled to NO_x_ reduction. Whether, perhaps, O isotopes rather than N isotopes might be a more sensitive tool for discerning reaction dynamics during heterotrophic denitrification coupled to Fe(II) oxidation, as suggested by Chen et al. (2020), remains unclear and should be investigated in the future. Moreover, Fe(II) oxidation, which occurred in parallel, may have impacted NO_2_^–^ reduction dynamics, but without marked influence on the N and O isotope fractionation. Taking the differences in N_2_O concentrations in consideration, the presence of Fe(II) seems to have affected cellular activity at least to some extent. While during heterotrophic growth, N_2_O was produced, but also fully consumed (except for the canonical denitrifier strain ATCC 19367), N_2_O accumulation appeared to be more frequent in the presence of Fe(II). Yet, all strains harbor the same *nosZ* gene, and since strain 2AN, for example, was still able to reduce N_2_O, inhibition of NosZ by Fe(II) can be excluded. This is further supported by previous NDFeO-based studies, which did not report any significant accumulation of N_2_O (and thus a possible inhibition of NosZ) (Kanaparthi et al., 2013; Ishii et al., 2016; Wang et al., 2016; Chen et al., 2018; Liu et al., 2018). Furthermore, considering that nitrite never accumulated in our experiments, inhibition of NosZ by nitrite (or free nitrous acid), as indicated by Perez-Garcia et al. (2017), is also very unlikely.

Future studies could focus on possible factors impacting N_2_O reduction in the presence of Fe(II) by using e.g., N_2_O/N_2_ isotope analysis. Since Fe(II) oxidation by nitric oxide (NO) can also not be excluded, a complete N budget in putative NDFeOx bacteria might help to shed light on the mechanistic details. Nevertheless, our results show that Fe(II)-amendment does not systematically impact N and O isotope fractionation, but rather yields isotope effects similar to those observed during heterotrophic growth. This may, in fact, not only support that the enzymatic control of NO_x_ reduction by putative NDFeOx bacteria is decoupled from Fe(II) oxidation, but also that Fe(II) oxidation is driven by another process instead.

## Conclusion

Here we tested whether a variation in short-chain organic acids and/or the presence of Fe(II), would impact N and O isotope fractionation dynamics during nitrate (NO_3_^–^) and nitrite (NO_2_^–^) reduction in laboratory denitrification experiments, and thus provide valuable insights into the mechanistic details of nitrate-dependent Fe(II) oxidation. To this end, putative nitrate-depending Fe(II)-oxidizing bacteria were cultivated under heterotrophic and mixotrophic growth conditions.

Our experiments revealed that the type of short-chained organic acids (acetate vs. succinate) had no obvious or systematic influence on N and O isotope fractionation dynamics during heterotrophic or mixotrophic NO_x_ reduction, despite some more general impacts on overall growth dynamics (e.g., reduced lag phases). In light of these results, the role of the carbon source in regulating the N and O isotope effects during NO_3_^–^ and NO_2_^–^ reduction remains elusive. We cannot exclude that more complex (i.e., cyclic or long-chained) organic carbon sources might in fact influence N and O isotope fractionation during NO_x_ reduction. The ^18^ε:^15^ε values calculated for heterotrophic (0.94 ± 0.01 ‰) and mixotrophic (0.96 ± 0.02 ‰) NO_3_^–^ reduction fall directly within the range reported for classical Nar-driven N and O isotope fractionation. However, under mixotrophic conditions, Fe(II) oxidation was incomplete and some strains did not grow. In addition, under mixotrophic conditions N_2_O accumulation was promoted, whereas under heterotrophic conditions, N_2_O did not accumulate in most strains. The enhanced N_2_O dynamics suggest an abiotic side reaction, however, the anticipated influence of a mere abiotic Fe(II) oxidation by NO_2_^–^ is also not indicated by the N isotopic evidence. In NO_2_^–^ reduction experiments, heterotrophic (^15^ε: 14.4 ± 3.2 ‰) and mixotrophic (^15^ε: 16.1 ± 1.4 ‰) growth conditions yielded similar values, again indicating that neither the organic substrate nor the presence of Fe(II) impacted N isotope fractionation dynamics. Hence, given that the isotope effects for both mixotrophic and heterotrophic experiments fall within the range observed for canonical organotrophic denitrification, we argue that neither nitrate nor nitrite reduction are directly linked to a purely enzyme-driven Fe(II) oxidation.

Although our results indicate that Fe(II) oxidation is decoupled from classical denitrification, we cannot exclude that Fe(II) oxidation either might be catalyzed by reactive surfaces (e.g., cell surface of denitrifying bacteria, minerals) or is even directly mediated by the presence of *c*-cytochromes present in excreted EPSs. Analyzing N_2_O and N_2_ isotope ratios in the future may help to elucidate, which intermediates are possibly involved in Fe(II) oxidation. In addition, labeling and/or abiotic experiments investigating sorption capacities and surface reactivity of cells, but also EPS, at environmentally relevant concentrations might be useful. This study, although providing valuable insights into N and O isotope fractionation dynamics during heterotrophic and mixotrophic NO_x_ reduction, illustrates some of the limitations of natural-abundance N and O isotope approaches with regards to constraining the mechanistic details behind Fe(II) oxidation in putative NDFeOx bacteria. Nevertheless, our data suggest that NDFeO in these cultures is indeed to some extent biologically induced/catalyzed (reactive surfaces/intermediates), but likely not directly coupled to the enzymatic-mechanism of NO_x_ reduction.

## Data availability statement

The raw data supporting the conclusions of this article will be made available by the authors, without undue reservation.

## Author contributions

A-NV, ML, and AK conceived the research project. A-NV designed and conducted all experiments. Isotope measurements as well as data analysis were performed by A-NV with help of, and under the supervision of, ML. CF gave valuable input with regards to structure and data presentation for this manuscript. A-NV, SW, and ML interpreted the data and prepared the manuscript with inputs from all other co-authors. All authors contributed to the article and approved the submitted version.
