# Supplementary material for: Unchanged nitrate and nitrite isotope fractionation during heterotrophic and Fe(II)-mixotrophic denitrification suggest a non-enzymatic link between denitrification and Fe(II) oxidation
Source: Front Microbiol. 2022 Sep 2;13:927475. doi: 10.3389/fmicb.2022.927475 (PMC9478938; doi:10.3389/fmicb.2022.927475)
Supplement: Supplementary file 1 [file Data_Sheet_1.pdf]

Unchanged nitrate and nitrite isotope fractionation during heterotrophic and Fe(II)-mixotrophic denitrification suggest a non-enzymatic link between denitrification and Fe(II) oxidation

Anna-Neva Visser[1,4*], Scott D. Wankel[2], Claudia Frey[1], Andreas Kappler[3,5], Moritz F. Lehmann[1,*]

[1]Aquatic and Isotope Biogeochemistry, Department of Environmental Sciences, Basel University, Basel, CH

[2]Stable Isotope Biogeochemistry, Marine Chemistry & Geochemistry, Woods Hole Oceanographic Institution, Woods Hole, Massachusetts, USA

[3]Geomicrobiology, Center for Applied Geosciences, Eberhard Karls University, Tübingen, DE

[4]now at: The Interuniversity Institute for Marine Sciences, Eilat & The Fredy and Nadine Herrmann Institute of Earth Sciences, Edmond J. Safra Campus, Givat Ram, Hebrew University of Jerusalem, Jerusalem, IL

[5]Cluster of Excellence: EXC 2124: Controlling Microbes to Fight Infection, Tuebingen, 72076, Germany

*** Correspondence:**
Corresponding Authors
annaneva.visser@mail.huji.ac.il, moritz.lehmann@unibas.ch

Supplementary Material

1 Supplementary Data

1.1 Supplementary Figures

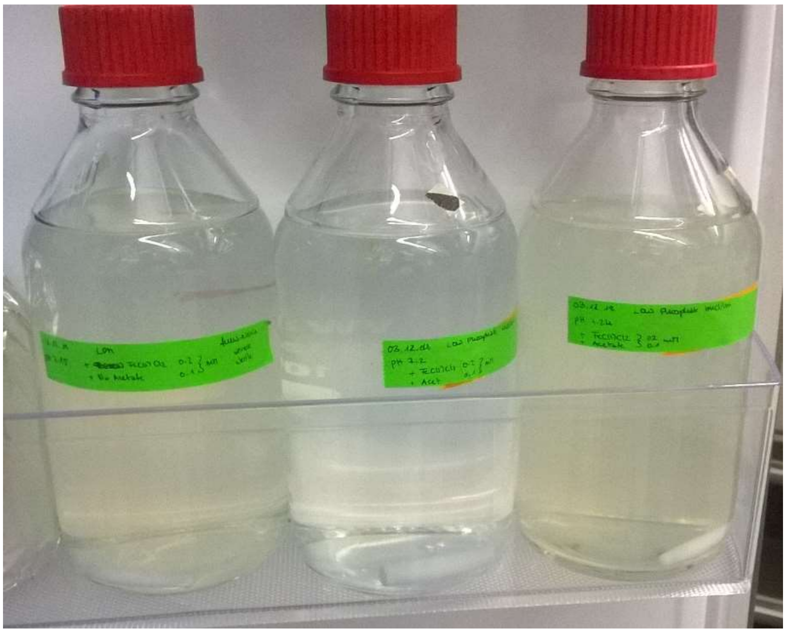

Supplementary Figure 1. Fe(II) precipitation after Fe(II)Cl_2 addition (0.2 mM) and storage at 4°C in the dark.

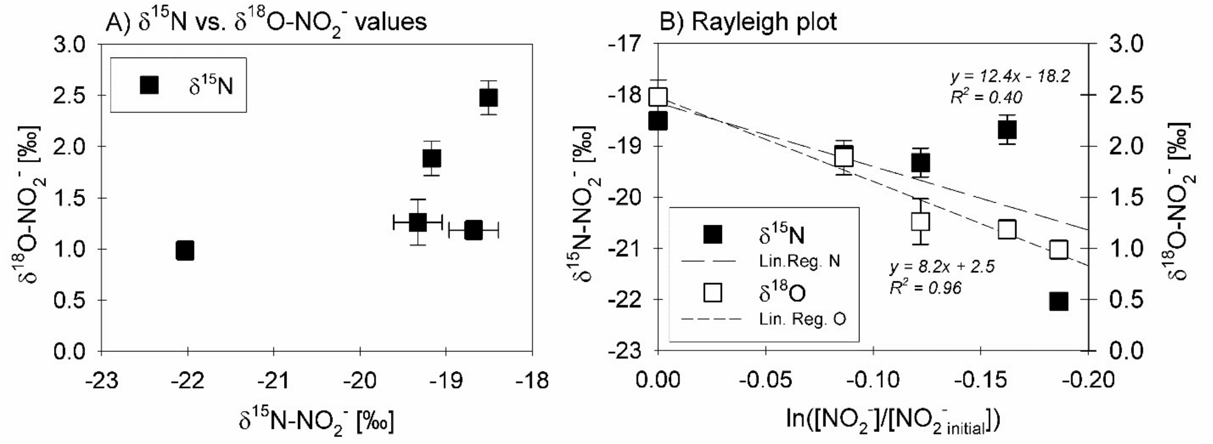

Supplementary Figure 2. $\delta^{18}O$ vs. $\delta^{15}N$-NO_2^- plot (A) and Rayleigh plot for $\delta^{15}N$ and $\delta^{18}O$-NO_2^- (B) for incubation of 2 mM NO_2^-, 2 mM Fe(II), and 1 mM acetate under abiotic conditions. A sacrificial sampling method was applied. The error bars represent standard error calculated from triplicates.

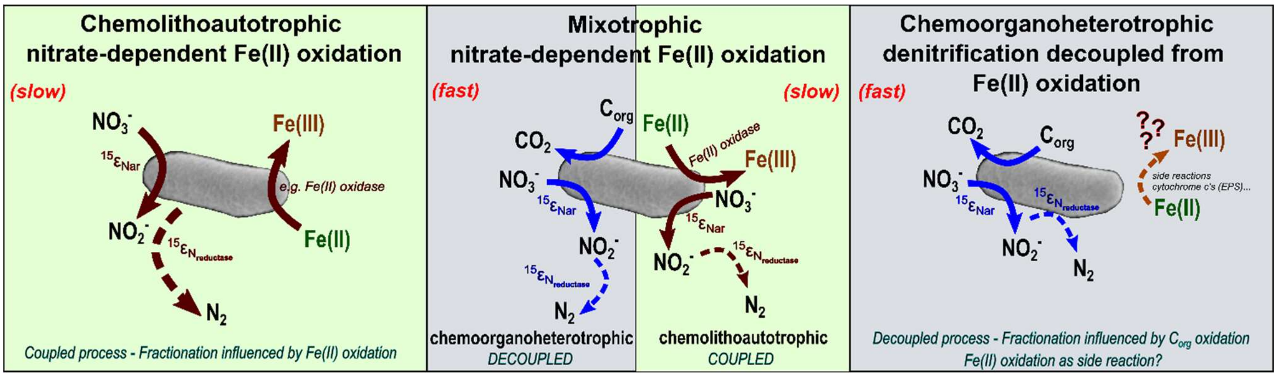

Supplementary Figure 3. Schematic illustration of the proposed processes and their possible influences on N isotope fractionation. Arrows with the same colour indicate direct coupling between reduction and oxidation steps presented. The direct coupling of nitrate reduction and Fe(II) oxidation (chemolithoautotrophic) is expected to result in lower energy yields and thus to be slower. In contrast, classical denitrification (chemoorganoheterotrophic) is known to obtain higher energy yields and thus to result in higher reaction rates. The different kinetics of each process should therefore result in dissimilar N and O isotope fractionation patterns. These differences are also expected to be observable, although less pronounced, if the organism switched between/ used (simultaneously) both pathways (mixotrophy).

1.2 Supplementary Tables

Supplementary Table 1: Overview of organisms used for N and O isotope fractionation experiments. Genomic analysis of all strains revealed the presence of genes encoding the respiratory nitrate reductase (Nar), as well as the heme (cd_1NIR)-containing nitrite reductase (NirS), and the nitrous oxide reductase (NosZ). Previous studies showed the capacity of the first three organisms to oxidize Fe(II) via nitrate reduction under mixotrophic conditions. Note that for strain BoFeN1, the genomic analysis also indicated the presence of the *nirK* gene, which is encoding for the Cu-containing nitrite reductase (NirK). Similarly, genomic analysis of strain 2002 indicated the presence of a *napA*-encoding gene.

Organism	Nitrate reductase	Nitrite reductase	N_2O reductase	Fe(II) oxidation	Sources
Acidovorax delafieldii strain 2AN	Nar	NirS	NosZ	mixotrophic	NCBI BioProject: PRJNA32605
Acidovorax sp. strain BoFeN1	Nar	NirS/(*nirK*)	NosZ	mixotrophic	(Gauger, 2016; Price et al., 2018)
Pseudogulbenkiania ferrooxidans strain 2002	Nar/(*napA*)	NirS	NosZ	mixotrophic	NCBI BioProject: PRJNA30761; (Byrne-Bailey et al., 2012)
Paracoccus denitrificans ATCC 19367*	Nar	NirS	NosZ	no	(Baumann et al., 1996)

* According to ATCC strains 19367, 13543, and 17741 may represent the same original isolate, https://www.lgcstandards-atcc.org/products/all/17741.aspx?geo_country=de#characteristics

1.3 NCBI sources for genome sequences (TRAP transporters)

Acidovorax strains NCBI e.g., Accession NZ_ACQT00000000.1
"Pseudogulbenkiania ferrooxidans" strain 2002 NCBI Accession: NZ_ACIS00000000.1
Paracoccus denitrificans strain ATCC 19367 e.g., NZ_CP035092.1
